# ^13^C-metabolic flux analysis of ethanol-assimilating *Saccharomyces cerevisiae* for *S*-adenosyl-l-methionine production

**DOI:** 10.1186/s12934-018-0935-6

**Published:** 2018-05-31

**Authors:** Kenshi Hayakawa, Fumio Matsuda, Hiroshi Shimizu

**Affiliations:** 1 Department of Bioinformatic Engineering, Graduate School of Information Science and Technology, Osaka University, 1-5 Yamadaoka, Suita, Osaka 565-0871 Japan; 2 KANEKA Fundamental Technology Research Alliance Laboratories, Graduate School of Engineering, Osaka University, 2-8 Yamadaoka, Suita, Osaka 565-0871 Japan; 3 Biotechnology Development Laboratories, Health Care Solutions Research Institute, Kaneka Corporation, 1-8 Miyamae-cho, Takasago-cho, Takasago, Hyogo 676-8688 Japan

**Keywords:** *Saccharomyces cerevisiae*, ^13^C-based metabolic flux analysis, Ethanol metabolism, Central carbon metabolism, Redox balance

## Abstract

**Background:**

*Saccharomyces cerevisiae* is a host for the industrial production of *S*-adenosyl-l-methionine (SAM), which has been widely used in pharmaceutical and nutritional supplement industries. It has been reported that the intracellular SAM content in *S. cerevisiae* can be improved by the addition of ethanol during cultivation. However, the metabolic state in ethanol-assimilating *S. cerevisiae* remains unclear. In this study, ^13^C-metabolic flux analysis (^13^C-MFA) was conducted to investigate the metabolic regulation responsible for the high SAM production from ethanol.

**Results:**

The comparison between the metabolic flux distributions of central carbon metabolism showed that the metabolic flux levels of the tricarboxylic acid cycle and glyoxylate shunt in the ethanol culture were significantly higher than that of glucose. Estimates of the ATP balance from the ^13^C-MFA data suggested that larger amounts of excess ATP was produced from ethanol via increased oxidative phosphorylation. The finding was confirmed by the intracellular ATP level under ethanol-assimilating condition being similarly higher than glucose.

**Conclusions:**

These results suggest that the enhanced ATP regeneration due to ethanol assimilation was critical for the high SAM accumulation.

**Electronic supplementary material:**

The online version of this article (10.1186/s12934-018-0935-6) contains supplementary material, which is available to authorized users.

## Background

*Saccharomyces cerevisiae* has been used in several industrial processes such as for the production of *S*-adenosyl-l-methionine (SAM) [1, 2]. SAM is synthesized from l-methionine and ATP in *S. cerevisiae*, and acts as a biological methyl group donor involved in many metabolic reactions such as transmethylation of proteins. The metabolite has been widely used in pharmaceutical and nutritional supplement industries [3]. For SAM production, *S. cerevisiae* strains used for Japanese sake brewing (Kyokai strains) are suitable owing to these higher intracellular contents among microorganisms [2]. The metabolic analysis showed that enhanced energy (ATP) regeneration in Kyokai strains contribute to high SAM production [4].

It has been reported that the SAM production was improved by feeding with ethanol. For instance, the Kyokai strains produce 10.8 g/L of SAM in a 10-L fermenter under ethanol feed conditions [5]. While the transcriptome analysis of *S. cerevisiae* grown on ethanol indicated the transcripts related to gluconeogenesis, the glyoxylate shunt, and the tricarboxylic acid (TCA) cycle were upregulated compared with those grown on glucose [6], the in vivo activity of the metabolic pathway involved in the SAM accumulation under ethanol-assimilating condition is still far from clear.

To further understand the mechanism in response to SAM production from ethanol, we performed a metabolic flux analysis for central carbon metabolism since SAM is biosynthesized from precursors to key intermediates including ribose 5-phosphate, ATP, oxaloacetate, and l-methionine using reductive power (NADPH) and chemical motive force (ATP). While a metabolic flux distribution in the ethanol-assimilating *S. cerevisiae* was estimated through metabolic flux balancing using a compartmented stoichiometric model in silico [6], the experimental determination by ^13^C-metabolic flux analysis (^13^C-MFA) is required to comprehensively quantify central carbon metabolism on ethanol in SAM-producing *S. cerevisiae* [7]. To our knowledge, there have been no prior ^13^C-MFA studies on ethanol metabolism in *S. cerevisiae*.

In the present study, ^13^C-MFA was conducted on a high-SAM-producing *S. cerevisiae* strain with ^13^C-labeled ethanol as the sole carbon source. ^13^C-MFA is based on the cultivation of the cells in medium containing ^13^C-labeled carbon sources. A metabolic flux distribution was estimated from the ^13^C-labeling patterns of intracellular metabolites using mass spectrometry. The selection of a suitable ^13^C-labeled carbon source is critical in the design of a ^13^C-MFA experiment since the accuracy of the flux estimation depends on the labeling patterns of the carbon source. However, a suitable experimental design remains unclear for the ^13^C-MFA of the central carbon metabolism in *S. cerevisiae* cultured with ethanol as the sole carbon source. Thus the design of the ^13^C-MFA experiment was optimized in this study by a computer simulation and found that 100% [2-^13^C] ethanol was the best carbon source for the precise measurements of metabolic fluxes. Using the optimized experimental design, the intracellular metabolic flux distribution of central carbon metabolism was successfully determined for the high SAM-producing *S. cerevisiae* strain (Kyokai no. 6) in the ethanol limited-chemostat culture. The results showed that the metabolic flux levels through the glyoxylate shunt and the later TCA cycle were upregulated during growth on ethanol, and the resultant activation of the oxidative phosphorylation should contribute the high SAM accumulation in the ethanol-assimilating conditions.

## Methods

### Strain and growth conditions

The *S. cerevisiae* strain used for Japanese sake brewing, Kyokai no. 6 (NBRC2346), was purchased from the National Biological Resource Center (NBRC, Chiba, Japan). This *S. cerevisiae* strain was cultivated in an aerobic carbon-limited chemostat culture with a working volume of 100 mL in a 250-mL fermentor (ABLE Co., Tokyo, Japan). The pH was maintained at 5.5 by the automatic addition of 1.0 N NaOH. The temperature was maintained at 30 °C. The stirring speed was 1000 rpm. The aeration rate was 200 mL/min. The dilution rate was 0.06 h^−1^. The synthetic medium used for cultivation contained 3.8 g/L ethanol, 5.0 g/L (NH_4_)_2_SO_4_, 0.50 g/L K_2_HPO_4_, and 3.4 g/L of yeast nitrogen base without amino acids and ammonium sulfate (Difco Laboratories, Franklin Lakes, NJ, USA). Vitamins and minerals were added to the medium, with the final composition as follows: 2.0 g/L KH_2_PO_4_, 1.5 g/L MgSO_4_, 2.5 mg/L ZnSO_4_, 2.4 mg/L MnSO_4_, 0.27 mg/L CuSO_4_, 0.20 g/L CaCl_2_, 4.0 mg/L FeCl_3_, 0.40 mg/L NaMoO_4_∙2H_2_O, 1.0 mg/L H_3_BO_3_, 0.20 mg/L KI, 0.20 g/L NaCl, 34 µg/L biotin, 1.6 mg/L Ca-pantothenate, 13 mg/L inositol, 7.8 mg/L thiamine-HCl, 2.3 mg/L pyridoxine-HCl, 0.40 mg/L para-aminobenzoic acid, 0.40 mg/L riboflavin, 0.80 mg/L niacin, and 4.0 µg/L folic acid. For ^13^C-MFA, the carbon source in the medium was replaced with [2-^13^C]ethanol. Samples taken from the reactor were centrifuged at 18,800×*g* for 5 min at 4 °C. The supernatant was used for analysis of extracellular metabolites. The cell pellet was used for ^13^C-labeling analysis by gas chromatography-mass spectrometry (GC–MS).

### Offline measurement

The analysis of SAM, cell dry weight (CDW), glucose, ethanol, glycerol and organic acids such as acetate were performed as described by Hayakawa et al. [4]. SAM concentration was measured by high-performance liquid chromatography (HPLC) LC2010A-HT (Shimadzu, Kyoto, Japan) after 10% HClO_4_ extraction. CDW was estimated using OD_600_ = 1 corresponding to 0.21 g_CDW_/L. Glucose and ethanol concentrations were measured enzymatically by using an analyzer (BF-7, Oji Scientific Instruments, Hyogo, Japan). An enzymatic kit was used for the quantification of glycerol (F-kit Glycerol, R-Biopharm, Washington, MO, USA) in the supernatant. The concentrations of acetate, pyruvate, succinate, fumarate, malate, citrate, and lactate in the supernatant were measured by HPLC (LC2010A-HT, Shimadzu).

The intracellular ATP, l-methionine, and SAM concentrations were measured using capillary electrophoresis time-of-flight mass spectrometry (CE-TOFMS) according to previously described methods [8]. Cultivated cells were harvested by filtration and washed with Milli-Q water. After the membrane was soaked in methanol solution containing an internal standard (H3304-1002, Human Metabolome Technologies, Yamagata, Japan), ultra-sonication was performed. After removal of the membrane, chloroform and water were added to the solution. The aqueous portion collected after mixing with a vortex mixer was filtered by Ultrafree MC-PLHCC 250 (Human Metabolome Technologies) and dried. For CE-TOFMS, pellets were suspended in the second internal standard (H3304-1004, Human Metabolome Technologies) solution. Samples were analyzed using the Agilent 7100 CE system with Agilent 6224 TOF–MS (Agilent Technologies, Santa Clara, CA, USA).

### Optimization of the ^13^C tracer for ^13^C-MFA by computational simulation

A computer simulation was conducted for the design of the ^13^C-MFA experiment as previously described [9]. The intracellular flux distribution data was determined using the modified literature data through metabolic flux balancing [6]. Data for the consumption and production rates were obtained in this study. A composition of [^13^C]ethanol was predetermined. The simulated ^13^C-enrichments were calculated for the 21 fragments of the following amino acids: (M−57)^+^ fragments for Ala, Asp, Glu, Gly, Phe, and Thr; (M−85)^+^ fragments for Ala, Asp, Glu, Gly, Ile, Leu, Pro, Thr, and Val; (M−159)^+^ fragments for Glu, Ile, Leu, Pro, and Val; and (M302)^+^ fragment for Asp; and then the Gaussian noise at 1% levels was added to produce hypothetical measurements for the ^13^C-enrichment data. A metabolic flux distribution was estimated by minimizing the residual between the hypothetical measurement and simulated ^13^C-enrichments of amino acid fragments. The 95% confidence intervals were determined using the grid search method [10, 11]. Precision scoring was calculated by the following equation to evaluate the range of the 95% confidence intervals:

1 $$S_{i} = \frac{{r_{i} }}{{r_{{\left[ {10:0:0:0} \right], i}} }}$$

where *r*_*i*_, *r*_[10:0:0:0], *i*_, and *S*_*i*_ are the range of the 95% confidence intervals, the range of 95% confidence intervals in ^13^C-labeled ethanol composition of 10:0:0:0 (100% non-labeled ethanol), and precision score for the *i*th flux. The maximum net flux was 500, which was normalized to an ethanol consumption rate of 100. The metabolic network shown in the following was used.

### Metabolic network

The metabolic model of *S. cerevisiae* for the ^13^C-MFA was based on previously published models, including glycolysis, the pentose phosphate (PP) pathway, the anaplerotic pathways, the TCA cycle, gluconeogenesis, and the transport reactions between the cytosol and mitochondria (Additional file 1: Tables S1 and S2) [4, 12, 13]. In the metabolic model, the reaction from the mitochondrial oxaloacetate and acetyl-CoA to isocitrate via citrate was assumed to be reversible. The amino acid biosynthesis pathways, including two pathways for glycine and alanine biosynthesis, were employed (Additional file 1: Table S2) [14]. The composition of the *S. cerevisiae* biomass was determined on basis of the literature (Additional file 1: Tables S3–S6) [15, 16]. Using these data and the stoichiometry of anabolic metabolism, the demands of precursor metabolites for cell growth were calculated (Additional file 1: Table S7) and used as constraint conditions in ^13^C-MFA (Additional file 1: Table S8).

### GC–MS analysis of proteinogenic amino acids

Cell pellets were hydrolyzed in 6 N HCl at 105 °C for 18 h. After removal of the debris by filtration, the hydrolysate was dried and dissolved in acetonitrile. For GC–MS, the hydrolysate was mixed with an equal volume of *N*-(*tert*-butyldimethylsilyl)-*N*-methyl-trifluoroacetamide (MTBSTFA), and the mixture was incubated at 95 °C for 1 h. Next, 1 μL of the sample was injected into the GC–MS system (7890A GC and 5975C GC/MSD, Agilent Technologies, Santa Clara, CA, USA), according to a previously described method [17, 18]. The GC–MS data were corrected considering the natural abundance of C, H, N, O, and Si isotopes for ^13^C-MFA [19]. The ^13^C-enrichments of the amino acid fragments at an isotopic steady state [χ(∞)] can be calculated from the following equation:

2 $$\chi \left( \infty \right) = \frac{{\chi (t) - e^{ - \mu t} \cdot \chi (0)}}{{1 - e^{ - \mu t} }} .$$

In Eq. (2), *t* represents the time that has elapsed since feeding ^13^C-labeled ethanol-containing medium, χ(*t*) and χ(0) are the ^13^C-enrichments of amino acid at *t* and start time since feeding the ^13^C-labeled ethanol-containing medium, respectively, and, µ is the dilution rate [20].

## ^13^C-metabolic flux analysis

The computational procedure for ^13^C-MFA was performed using a Python version of OpenMebius implemented in Python 2.7.9 [9, 21], by which 22 independent fluxes were iteratively tuned by minimizing the residual between the experimental and simulated ^13^C-enrichments of proteinogenic amino acid fragments. Furthermore, the ^13^C-labeling patterns of CO_2_ were independently optimized in the flux estimation. Amino acid fragments used for the flux optimization were shown above. Nonlinear optimization was performed using the SLSQP (sequential least squares programming) function implemented in PyOpt 1.2 [22]. The 90% confidence intervals were determined using the grid search method [10, 11].

### NADPH and ATP demands for cell growth and SAM production

The NADPH demands for cell growth in assimilating glucose and ethanol were calculated by using Additional file 1: Tables S3–S6 and the KEGG database (http://www.genome.jp/kegg/) (9.42 and 11.95 mmol/g_CDW_, respectively). The literature data were used for the ATP demands for cell growth on glucose and ethanol (39.78 and 105.56 mmol/g_CDW_, respectively) [23]. A non-growth-associated maintenance ATP requirement of 1.0 mmol ATP/g_CDW_/h was also considered [24]. The total ATP cost for SAM biosynthesis was found to be 13.0 mol ATP per mol SAM [4].

## Results

### Physiological parameters of ethanol-assimilating *S. cerevisiae*

The *S. cerevisiae* strain used for Japanese sake brewing (Kyokai no. 6, NBRC2346) was cultivated in an ethanol-limited chemostat culture under aerobic condition at a dilution rate of 0.06 h^−1^. The physiological parameters in the cultivation are shown in Table 1. A comparison with the previous glucose-limited chemostat culture [4] showed that the SAM specific production rate and intracellular content in assimilating ethanol (0.27 μmol/g_CDW_/h and 1.8 mg/g_CDW_, respectively) were greater than that of a glucose-limited culture under the same dilution and carbon atom supply rate conditions (0.089 μmol/g_CDW_/h and 0.58 mg/g_CDW_, respectively). The biomass yield in assimilating ethanol was lower than that of glucose as with previous research [25, 26]. In contrast, the acetate yield in assimilating ethanol was higher than that of glucose. This might be related to the enhanced expression of acetaldehyde dehydrogenase (*ALD4* and *ALD6*) under assimilating ethanol conditions [6].

**Table 1:** Fermentation profiles of the high-SAM-producing strain (Kyokai no. 6) under aerobic carbon-limited chemostat cultures (dilution rate of 0.06 h^−1^)

	Carbon source	
	Glucose^a^	Ethanol
Carbon source consumption rate (mmol/g_CDW_/h)	0.65	2.5
SAM content (mg/g_CDW_)	0.58	1.8
Yield on carbon source		
Cell (g_CDW_/mol C)	15	12
CO_2_ (mol C/mol C)^b^	0.41	0.54
Specific production rate (μmol/g_CDW_/h)		
SAM	0.089	0.27
Malate	3.6	5.1
Acetate	4.9	29
Lactate	5.9	2.0
Pyruvate	0	0.27
Citrate	1.3	0.51
Succinate	4.2	4.3
Glycerol	0.35	0.44
Ethanol	0	–

All data were obtained from a single experiment. *CDW* cell dry weight

^a^Data from previous study [4]

^b^Estimated by the carbon recoveries for biomass synthesis calculated from the specific growth rate and the reported elemental composition of carbon (glucose: 0.455 g/g_CDW_, ethanol: 0.467 g/g_CDW_) [15], and by the specific rates for carbon source consumption and for products including SAM productions. The total carbon recovery was considered to be 1.0

### Design of ^13^C-MFA using ^13^C-labeled ethanol

As mentioned in the background, ^13^C-MFA is based on the cultivation of cells in medium containing ^13^C-labeled carbon sources. Selection of a suitable ^13^C-labeled carbon source is critical in the design of a ^13^C-MFA experiment since the accuracy of the flux estimation depends on labeling patterns of the carbon source. In this study, a suitable experiment was designed for the ^13^C-MFA of the central carbon metabolism in *S. cerevisiae* cultured on ethanol as sole carbon source. A relationship between the composition of ^13^C-labeled ethanol including [1-^13^C], [2-^13^C], and [U-^13^C]ethanol, and 95% confidence intervals of the estimated flux levels were investigated by a computer simulation of ^13^C-MFA experiment.

The metabolic model of *S. cerevisiae* describing the stoichiometry equations and carbon atom transitions of each metabolic reaction was constructed from previously published models with modifications (Additional file 1: Tables S1 and S2) [4, 12, 13]. The computer simulation was conducted by the following procedure [9]: using a flux distribution of ethanol-assimilating *S. cerevisiae* estimated by an in silico calculation method [6], a series of artificial mass spectra datasets of amino acid fragments were generated for various compositions of [^13^C]ethanol (all patterns of non-labeled, [1-^13^C], [2-^13^C], and [U-^13^C]ethanol with a 50% step size) by adding Gaussian noise at 1% levels. The metabolic flux distribution and 95% confidence intervals were determined using the artificial mass spectra datasets by the ^13^C-MFA procedure (Additional file 1: Table S9). A precision score *S*_*i*_ was determined for each metabolic reaction *i* from the data of the 95% confidence intervals. Useful compositions of [^13^C]ethanol were investigated by comparing the precision score *S*_*i*_ of estimated flux distributions.

**Fig. 1:**
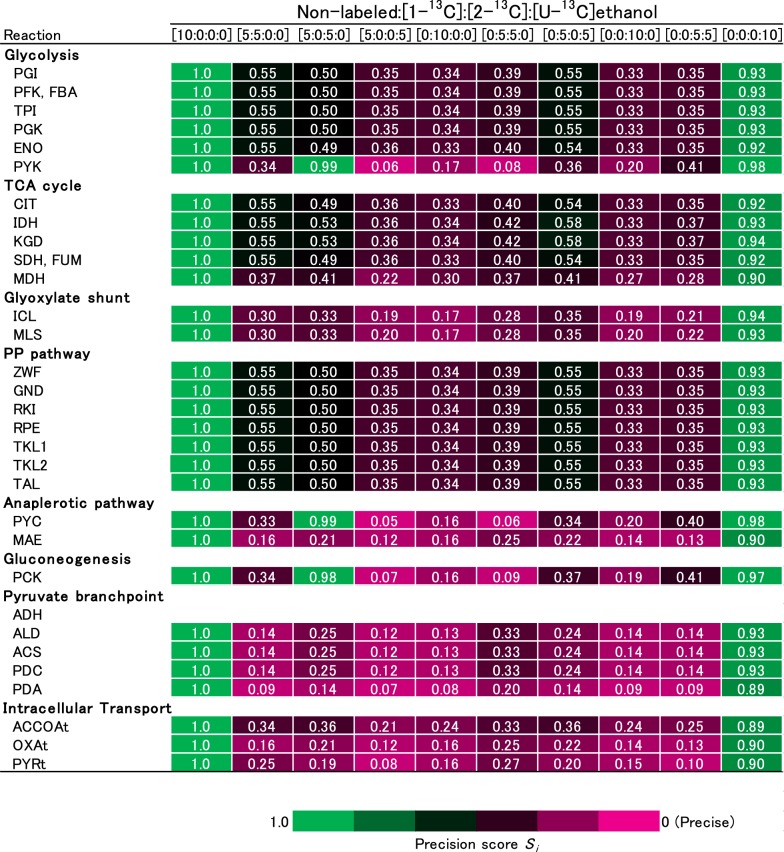
Heatmap of the precision score *S*_*i*_ levels estimated by the computer simulation of ^13^C-MFA using 10 mixtures of non-labeled, [1-^13^C], [2-^13^C], and [U-^13^C]ethanol as the carbon sources. The magenta and green colors in the boxes represent narrower (better precision) and wider (poorer precision) 95% confidence interval levels of the estimated metabolic fluxes on each reaction, respectively

For example, the 95% confidence intervals of isocitrate dehydrogenase (IDH) flux (isocitrate→α-ketoglutarate + CO_2_) with mixtures of non-labeled, [1-^13^C], [2-^13^C], [U-^13^C]ethanol at 10:0:0:0, 5:5:0:0, and 0:0:10:0 were estimated to be 2.5–45.1, 19.0–42.3, and 27.4–41.6, from which *S*_*IDH*_ were estimated to be 1.0, 0.55, and 0.33, respectively (Additional file 1: Table S9). Since a smaller *S*_*IDH*_ score shows a more precise flux estimation, the results indicated that the mixture of non-labeled, [1-^13^C], [2-^13^C], [U-^13^C]ethanol at 0:0:10:0 was a suitable carbon sources for the flux estimation of this reaction in the three mixtures. Figure 1 shows a heatmap in the precision score *S*_*i*_ based on the range of 95% confidence interval for the estimated flux in each composition of ^13^C-labeled ethanol. The magenta and green colors represent narrower (better precision) and wider (poorer precision) 95% confidence intervals, respectively. This result indicates that the range of the 95% confidence interval depended greatly on the composition of ^13^C-labeled ethanol. The mixture ratio at 0:0:10:0 (100% [2-^13^C]ethanol) showed the smallest sum of the precision score *Si* in the TCA cycle and PP pathway among all ^13^C-labeling conditions (Fig. 1). Based on the results, [2-^13^C]ethanol was employed for the ^13^C-MFA of the central carbon metabolism in *S. cerevisiae* cultured on ethanol as the sole carbon source.

## ^13^C-Metabolic flux analysis

The Kyokai no. 6 strain was cultivated for ^13^C-MFA by an ethanol-limited chemostat culture under aerobic conditions at a dilution rate 0.06 h^−1^. At 98 h after the start of cultivation when the cell growth reached steady-state, the medium containing 100% [2-^13^C]ethanol was fed to the chemostat cultures. The biomass samples were harvested at 1.3, 2.3, 2.7, 3.7, 4.1, and 5.1 residential times after feeding ^13^C-labeled ethanol. Following acid hydrolysis and derivatization, ^13^C-enrichment of proteinogenic amino acids was measured using GC–MS analysis (Additional file 1: Fig. S1). The intracellular metabolism might reach an isotopic steady-state after an infinite amount of time. In this study, the ^13^C-enrichment data indicated by Eq. (2) were used to calculate metabolic flux distributions (Additional file 1: Table S10 and Fig. S1).

A metabolic flux distribution was estimated by minimizing the difference between the computationally simulated ^13^C-enrichments of proteinogenic amino acids and the experimentally obtained data by GC–MS. The differences (residual sum of squares) between experimental and simulated ^13^C-enrichment data for the best-fitted metabolic flux distributions that passed the χ^2^-test (α = 0.05) were small (69.5) [11], indicating that the estimated flux distributions could explain the experimentally obtained ^13^C-enrichments (Additional file 1: Table S10). Based on the best-estimate metabolic flux distribution, 90% confidence intervals of the metabolic flux levels were determined using the grid search method [10, 11].

**Fig. 2:**
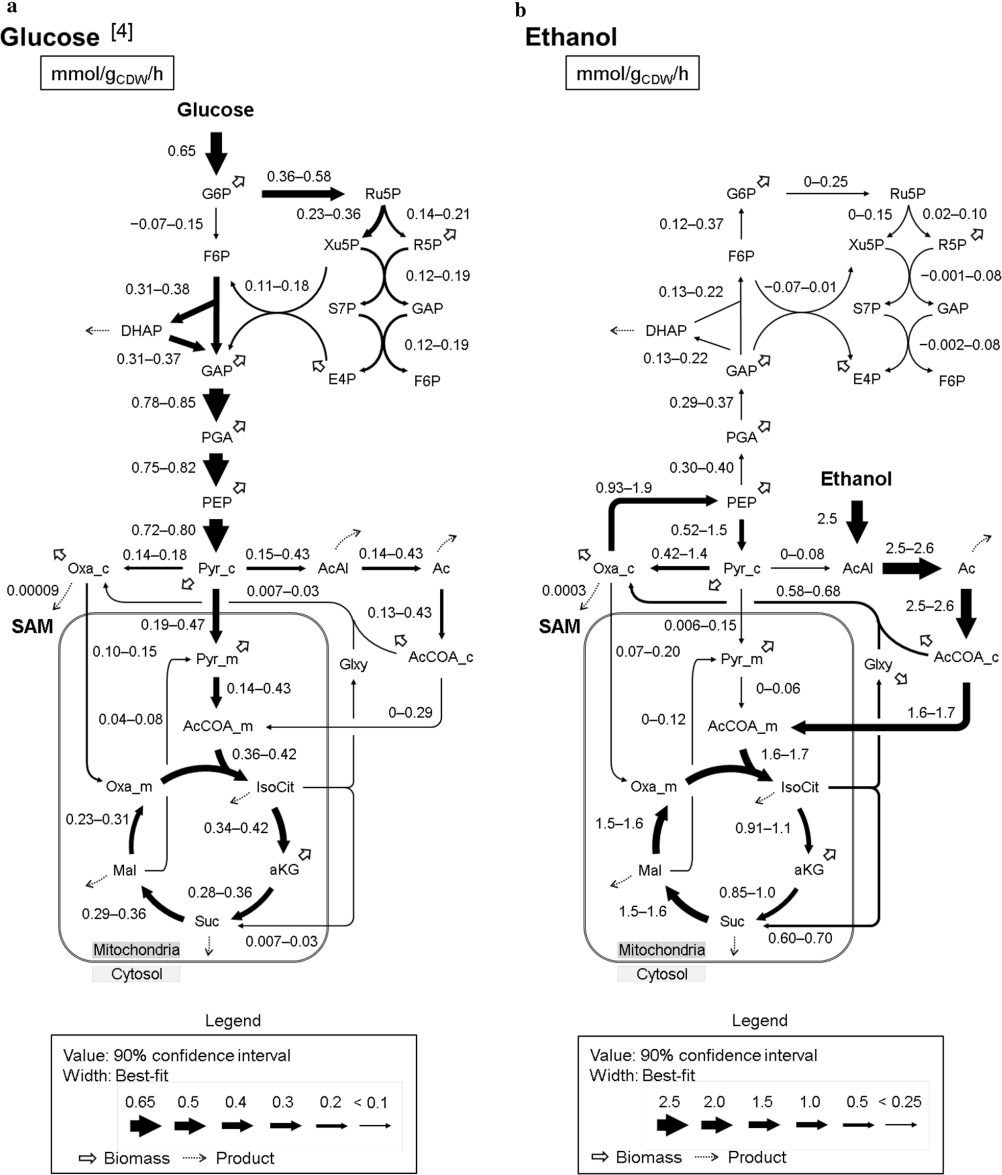
Metabolic flux distributions (mmol/g_CDW_/h) for *S. cerevisiae* grown on glucose (**a**) [4] and ethanol (**b**) at a dilution rate of 0.06 h^−1^. Flux values in the central carbon metabolism are shown with 90% confidence intervals. The width of each arrow indicates best-fit value (Additional file 1: Table S11). The anabolic reactions from metabolic intermediates to biomass are represented by small arrows. The product synthesis reactions are represented by dotted arrows. These flux values are shown in Additional file 1: Table S8. *G6P* glucose-6-phosphate, *F6P* fructose-6-phosphate, *DHAP* dihydroxyacetone phosphate, *GAP* 3-phosphoglyceraldehyde, *PGA* 3-phosphoglycerate, *PEP* phosphoenolpyruvate, *Pyr_c* cytosolic pyruvate, *Pyr_m* mitochondrial pyruvate, *AcCOA_c* cytosolic acetyl-CoA, *AcCOA_m* mitochondrial acetyl-CoA, *AcAl* acetaldehyde, *Ac* acetate, *IsoCit* isocitrate, *aKG* α-ketoglutarate, *Suc* succinate, *Mal* malate, *Oxa_c* cytosolic oxaloacetate, *Oxa_m* mitochondrial oxaloacetate, *6PG* 6-phosphogluconate, *Ru5P* ribulose-5-phosphate, *R5P* ribose-5-phosphate, *Xu5P* xylulose-5-phosphate, *S7P* sedoheptulose-7-phosphate, *E4P* erythrose-4-phosphate, *Glxy* glyoxylate

The 90% confidence intervals of the flux levels on glucose and ethanol in the Kyokai no. 6 strain at a dilution rate 0.06 h^−1^ are shown in Fig. 2. The results for glucose have been previously published [4]. The results revealed that the metabolic flux distribution was totally rewired in the ethanol culture from that under the glucose culture. The Embden–Meyerhof–Parnas (EMP) pathway was reversed from the glycolytic direction in the glucose culture to the direction for gluconeogenesis because the assimilated ethanol flowed into the EMP pathway via the glyoxylate shunt and then converted to glucose-6-phosphate. The metabolic flux in the glyoxylate shunt (IsoCit→Glxy + Suc, AcCOA_c + Glxy→Oxa_c) for the glucose and ethanol assimilations were 0.007–0.03 and 0.58–0.68 mmol/g_CDW_/h, respectively. This was in agreement with the gene transcription levels of *ICL1*, *ICL2*, *MLS1*, and *MLS2* in the glyoxylate shunt that were upregulated in a transcriptome analysis of *S. cerevisiae* grown on ethanol described previously [6]. Since the expression of malate synthase (AcCOA_c + Glxy→Oxa_c) was essential for growth on ethanol [27], the result confirmed that the pathway was active during the assimilation of ethanol.

**Fig. 3:**
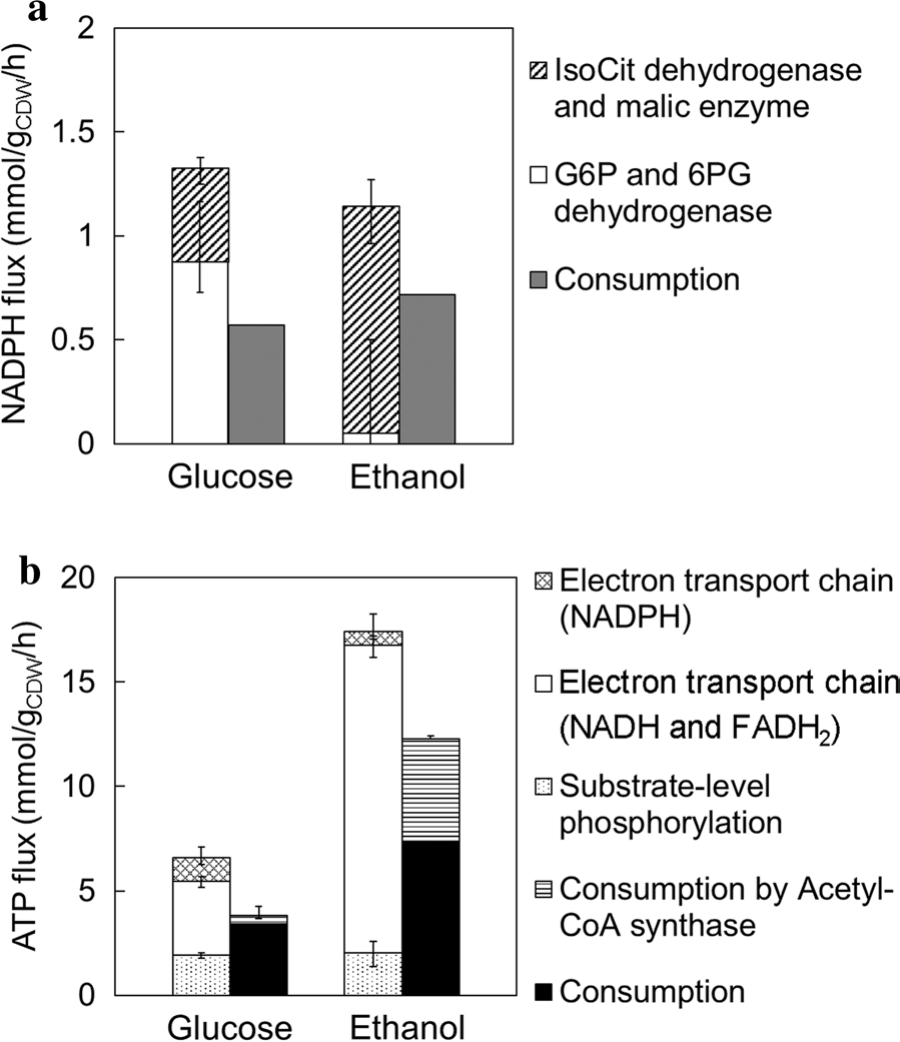
Redox and energy balances. NADPH (**a**) and ATP (**b**) balances in assimilating glucose and ethanol were determined at a dilution rate of 0.06 h^−1^. The error bars at each bar represent the 90% confidence intervals of the flux estimations

To investigate the cofactor balance under the ethanol-assimilating conditions, metabolic flux levels responsible for the NADPH and ATP regeneration were compared (Fig. 3a, b). In *S. cerevisiae*, NADPH was mainly regenerated via the PP pathway (glucose-6-phosphate dehydrogenase and phosphogluconate dehydrogenase), malic enzyme, and isocitrate dehydrogenase. The results showed that the TCA cycle functioned as the main provider of NADPH on ethanol since the flux level of isocitrate dehydrogenase (IsoCit + NADP^+^→aKG + NADPH + CO_2_) in the TCA cycle was higher on ethanol (0.91–1.1 mmol/g_CDW_/h) compared to glucose (0.34–0.42 mmol/g_CDW_/h) (Figs. 2 and 3a). On the other hand, the glucose-6-phosphate dehydrogenase and phosphogluconate dehydrogenase flux (G6P + 2 NADP^+^→Ru5P + 2 NADPH + CO_2_) in the oxidative PP pathway was 0–0.25 mmol/g_CDW_/h on ethanol, which was lower compared to the 0.36–0.58 mmol/g_CDW_/h on glucose, indicating that the carbon fluxes of the central carbon metabolism on ethanol were redirected into the TCA cycle (Fig. 2). The malic enzyme flux (Mal + NADP^+^→Pyr_m + NADPH + CO_2_) was similar on glucose and ethanol (0.04–0.08 and 0–0.12 mmol/g_CDW_/h, respectively), which showed that this reaction did not contribute greatly in the regeneration of NADPH on both carbon sources (Fig. 2).

Figure 3b shows the ATP balance in the glucose- and ethanol-assimilating conditions. The data revealed that oxidative phosphorylation using the electron transport system during respiration was a major source of ATP regeneration. The metabolic flux distribution showed that the NADH supply for the electron transport system are from the EMP pathway, TCA cycle, glyoxylate shunt, alcohol dehydrogenase, and aldehyde dehydrogenase. The flux levels of these reactions were also largely different between glucose and ethanol. For instance, the malate dehydrogenase flux (Mal + NAD^+^→Oxa_m + NADH) in the TCA cycle on ethanol corresponded to 59–65% of ethanol consumption rate (1.5–1.6 mmol/g_CDW_/h), while the flux level on glucose corresponded to 35–48% of glucose consumption rate (0.23–0.31 mmol/g_CDW_/h) (Fig. 2). In contrast, the pyruvate dehydrogenase (Pyr_m + NAD^+^→AcCOA_m + CO_2_ + NADH) in the EMP pathway was inactive on ethanol because of a low flux (0–0.06 mmol/g_CDW_/h), indicating that the TCA cycle also functioned as the provider of NADH on ethanol (Fig. 2). Therefore, this result suggests that the high TCA activity in ethanol assimilation caused the lower cell yield on carbon source compared with glucose assimilation, since two CO_2_ molecules were released from the TCA cycle (Table 1).

Next, the carbon flows around cytosolic oxaloacetate, which is the precursor for SAM, were compared. The levels of SAM biosynthesis flux from cytosolic oxaloacetate on glucose- and ethanol-assimilating (0.09 and 0.3 µmol/g_CDW_/h, respectively) were only 0.01% or less of the glucose- and ethanol-consumption flux values (0.65 and 2.5 mmol/g_CDW_/h, respectively) (Fig. 2). In turn, the flux levels of pyruvate carboxylase (Pyr_c + CO_2_→Oxa_c) on glucose and ethanol were 0.14–0.18 and 0.42–1.4 mmol/g_CDW_/h, respectively, indicating that pyruvate carboxylase were used to supply cytosolic oxaloacetate on both carbon sources (Fig. 2). This result was confirmed by the detected enzyme activity under the ethanol-grown culture [26].

### Redox balance

As mentioned above, ^13^C-MFA showed that the flux levels related to NADPH and ATP regeneration were largely different during growth on glucose and ethanol, while the flux of cytosolic oxaloacetate to SAM biosynthesis corresponded to 0.01% of the ethanol consumption flux value. Hence, the relationship between cofactor (NADPH and ATP) balances and SAM biosynthesis was investigated. To analyze the NADPH balance, the NADPH regeneration rates estimated from the metabolic flux data were compared with the NADPH consumption rates for cell growth. Since the composition of *S. cerevisiae* biomass differs among the carbon source used for the culture, NADPH demand levels were determined using Additional file 1: Tables S3–S6 and the KEGG database (see Materials and methods). As shown in Fig. 3a, NADPH regeneration rates were larger than the consumption rates for cell growth in both conditions. In assimilating glucose and ethanol, the differences between NADPH regeneration and consumption rates were 0.75 and 0.42 mmol/g_CDW_/h, respectively, indicating that the difference on glucose was greater than that of ethanol (Fig. 3a). Excess NADPH was assumed to be used for ATP production by the oxidation of NADPH as previously shown [4].

### ATP balance

Since the high levels of SAM production by the Kyokai no. 6 strain could be attributed to enhanced ATP regeneration [4], ATP balances for regeneration and consumption were investigated (Fig. 3b). Total ATP regeneration rates were calculated based on the metabolic flux of reactions responsible for the respiration and substrate level phosphorylation. The biosynthetic ATP demand was estimated from the literature data (see “Methods”). The ATP consumption rate for cytosolic acetyl-CoA synthesis was calculated from acetyl-CoA synthase flux (Ac→AcCOA_c). The difference between ATP regeneration and consumption on ethanol was 5.1 mmol/g_CDW_/h, indicating that the difference on ethanol was 1.9-fold greater than that of glucose (2.8 mmol/g_CDW_/h) (Fig. 3b).

### Intracellular ATP, l-methionine, and SAM concentration

**Fig. 4:**
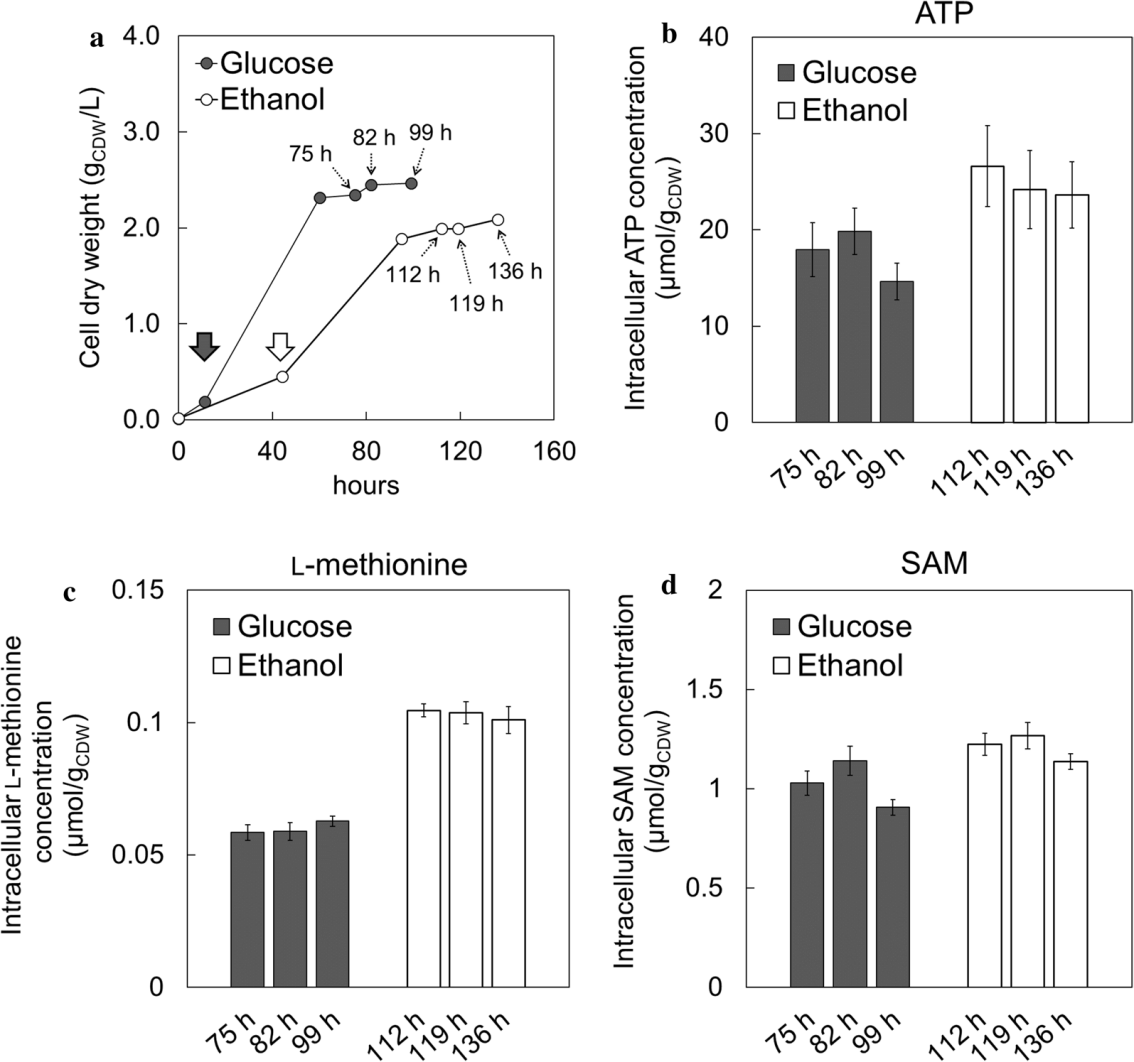
Carbon-limited chemostat cultures of *S. cerevisiae*. **a** Cell dry weight (CDW; g_CDW_/L); **b** intracellular ATP concentration (µmol/g_CDW_); **c** intracellular l-methionine concentration (µmol/g_CDW_); **d** intracellular SAM concentration (µmol/g_CDW_). The bold arrows indicate the time points at the start of the carbon-limited chemostat cultures. The dash arrows represent the time points of sampling for intracellular metabolite analysis. Values of intracellular ATP concentration represent the average of five measurements with error bars calculated as the standard deviations of the means. Values of intracellular l-methionine and SAM concentration represent the average of three measurements with error bars calculated as the standard deviations of the means

The ^13^C-metabolic flux analysis revealed that SAM accumulation on ethanol should be derived from enhanced ATP regeneration. It was also expected that the intracellular ATP concentration on ethanol could be higher than that of glucose since the SAM content in assimilating ethanol at a dilution rate of 0.06 h^−1^ (1.8 mg/g_CDW_) (Table 1) was greater than that of glucose at a dilution rate of 0.1 h^−1^ (1.0 mg/g_CDW_) [4]. Then, intracellular ATP concentrations were determined by CE-TOFMS analysis to further investigate the effects of ATP levels on SAM productivity. For the comparison of ATP levels, glucose- and ethanol-limited chemostat cultures were conducted at dilution rates of 0.1 and 0.06 h^−1^, respectively. After the batch phase (at 11 and 44 h after the initiation of cultivation for glucose and ethanol, respectively), the carbon-limited chemostat cultures were started. After reaching steady-state cell growth, the *S. cerevisiae* cells were repeatedly sampled (at 75, 82, and 99 h on glucose; and at 112, 119, and 136 h on ethanol; respectively) (Fig. 4a). In this study, three samples were considered to be experimental triplicate. The intracellular ATP levels in the cells grown on ethanol (24.8 ± 1.6 μmol/g_CDW_) was 1.4-fold higher than that of glucose (17.5 ± 2.6 μmol/g_CDW_) (Fig. 4b). This result confirmed that the *S. cerevisiae* cells grown on ethanol had the potential for high SAM content due to high intracellular ATP levels as shown in the metabolic flux distribution estimated using ^13^C-MFA. Similarly, the intracellular l-methionine and SAM levels on ethanol (0.10 ± 0.0019 μmol/g_CDW_ and 1.2 ± 0.067 μmol/g_CDW_) were 1.7- and 1.2-fold higher than those of glucose (0.060 ± 0.0024 μmol/g_CDW_ and 1.0 ± 0.12 μmol/g_CDW_), respectively, indicating that improvement of intracellular ATP and l-methionine levels enhanced SAM biosynthesis (Fig. 4c, d).

## Discussion

In the present study, ^13^C-MFA was conducted to investigate a metabolic regulation responsible for the high intracellular SAM content in ethanol-assimilating *S. cerevisiae* Kyokai no. 6 strain [5, 28]. The design of the ^13^C-MFA experiment was optimized by computer simulations, which showed that 100% [2-^13^C]ethanol was the best carbon source for the precise estimation of metabolic fluxes through the TCA cycle and PP pathway (Fig. 1 and Additional file 1: Table S9). An advantage of having 100% [2-^13^C]ethanol in ^13^C-MFA was that the second carbon of ethanol contributes to the generation of various metabolic intermediates with various ^13^C-labeling pattern, which in turn increases the sensitivity to fluxes. On the other hand, the first and fourth carbons of mitochondrial oxaloacetate derived from the first carbon of ethanol are released as CO_2_ in the TCA cycle [29]. The ^13^C-MFA in *Escherichia coli* cultured on acetate was performed using the mixtures of non-labeled, [2-^13^C]acetate at a ratio of 8:2 because of the same reason [30].

A comparison of the metabolic flux distribution on glucose and ethanol revealed that the metabolic flux through the EMP pathway was reversed by the activation of the glyoxylate shunt and gluconeogenesis during growth on ethanol (Fig. 2). The metabolic redirection coincided with the regulation in mRNA and protein expression levels as reported in previous studies. It has been reported that *S. cerevisiae* grown on ethanol increased the transcript levels of genes involved in gluconeogenesis and the glyoxylate shunt compared to cells grown on glucose [6]. Furthermore, comparable results have been observed in a proteome analysis under chemostat cultures limited for glucose and ethanol [31]. The previous transcriptome and proteome studies during the diauxic shift in *S. cerevisiae* [32–34] also showed that the glyoxylate shunt, gluconeogenesis, and oxidative phosphorylation were activated after starting the assimilation of the produced ethanol.

On the other hand, the downregulation of the pyruvate dehydrogenase flux level (Pyr_m→AcCOA_m + CO_2_) at the entry point of the TCA cycle disagreed with a previous transcriptome analysis because the expression levels of *PDA1*, *PDB1*, *PDX1*, and *LPD1* genes in the ethanol-assimilating conditions increased from that of glucose (Fig. 2) [6]. This may have been due to pyruvate dehydrogenase being allosterically regulated by NADH [35]. These results suggested that the fluxes through the central carbon metabolism in *S. cerevisiae* were regulated at the transcript, translational, and post-translational levels [6, 31, 36].

**Fig. 5:**
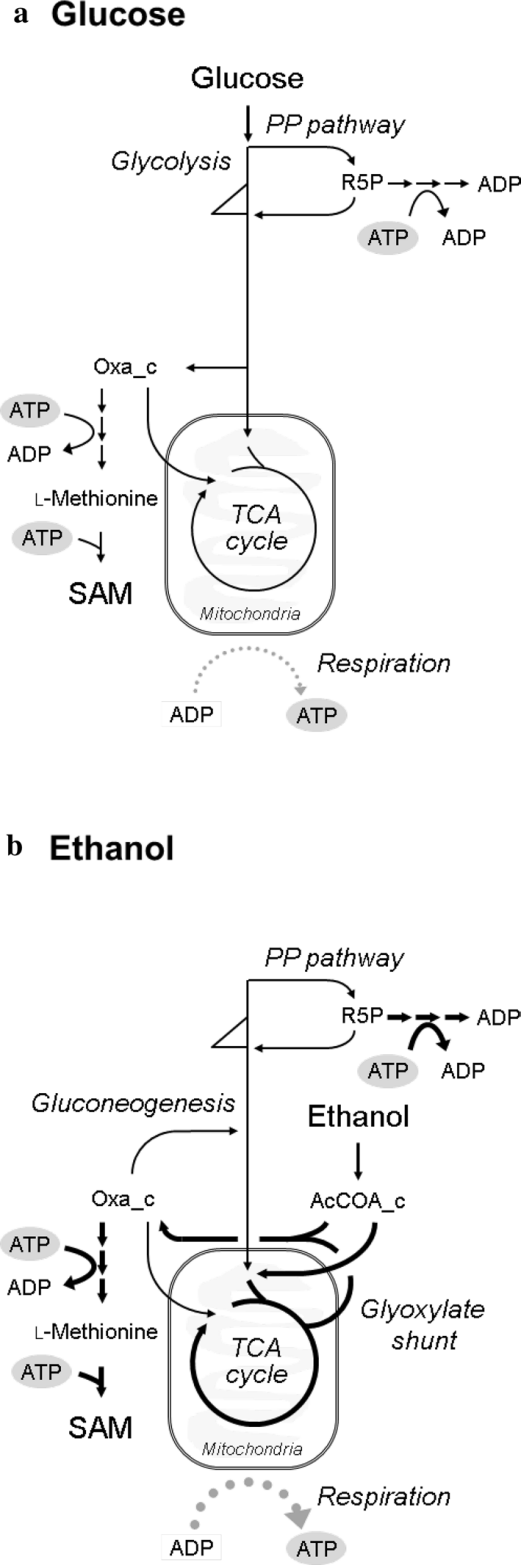
Hypothesized mechanism for the high SAM accumulation. **a** Glucose; **b** ethanol (carbon source for higher SAM accumulation). The dotted gray arrows represent respiration. The ^13^C-MFA results revealed that high SAM accumulation can be explained by enhanced ATP regeneration with high respiration activity

It has been reported that the ethanol-assimilating *S. cerevisiae* cells showed a higher oxygen uptake rate [15]. The results of ^13^C-MFA confirmed that ATP regeneration via the oxidative phosphorylation significantly increased under the ethanol-assimilating conditions (Fig. 5). Furthermore, estimation of the ATP balance revealed that a higher amount of excess ATP was produced on ethanol (Fig. 3b), since upregulation of ATP regeneration via the oxidative phosphorylation was greater than the increase in ATP consumption for gluconeogenesis and cytosolic acetyl-CoA synthesis during ethanol assimilation [25]. These results suggested that the excess ATP should activate SAM biosynthesis. However, the excess ATP regeneration flux (5.1 mmol/g_CDW_/h) was clearly greater than the ATP consumption flux for SAM production (3.5 µmol/g_CDW_/h) (Table 1 and Fig. 3b). The excess ATP may be utilized for the response of stress caused by acetaldehyde and acetate [37, 38].

The analysis of the intracellular metabolites (Fig. 4) revealed that the ATP level in the cells grown on ethanol was higher than that of glucose (Fig. 4b). It was reported that the increase of intracellular ATP level enhanced the SAM production in *S. cerevisiae*. The addition of sodium citrate improved the isocitric acid dehydrogenase activity and ATP level in the cell, which promoted the conversion of methionine into SAM [39]. Optimization of the culture medium also revealed that the restriction of cell growth, and the enhancement of the intracellular ATP level and SAM production were achieved by reducing the supplemented yeast extract [8]. Moreover, the result of this study that the intracellular l-methionine level in ethanol assimilating was higher than that of glucose (Fig. 4c). Since l-methionine biosynthesis consumes cytosolic acetyl-CoA in *S. cerevisiae* [40], the upregulation might be concerned with the higher flux level of acetyl-CoA synthase (Ac→AcCOA_c) on ethanol (2.5–2.6 mmol/g_CDW_/h) compared to glucose (0.13–0.43 mmol/g_CDW_/h) (Fig. 2). These results indicated that both ATP and l-methionine levels in cells had a positive effect on SAM biosynthesis (Table 1 and Fig. 4d).

In *S. cerevisiae*, cytosolic acetyl-CoA was synthesized from acetate and CoA by acetyl-CoA synthase with consuming ATP [41]. The increased ATP regeneration in the ethanol assimilation conditions would also enhance the production of other useful chemicals, since the cytosolic acetyl-CoA is a common precursor for the biosynthesis of fatty acid ethyl ester (biodiesel), geraniol (flavor), and amorpha-4, 11-diene (precursor to artemisinin) [42–44]. The results of this study also suggested that the saving on the ATP consumption for cytosolic acetyl-CoA would further increase SAM production. For instance, it has been reported that introducing the metabolic reaction that produces acetyl-CoA from acetaldehyde using an ATP-independent enzyme could increase cell yield [45].

The ^13^C-MFA conduced in this study revealed that excess ATP regeneration via the activation of oxidative phosphorylation was a mechanism responsible for the SAM overproduction under the ethanol-assimilating conditions. A detailed mechanism for the metabolic redirection in the central carbon metabolism could be uncovered by a systems-level analysis of the metabolism in ethanol-assimilating *S. cerevisiae* using ^13^C-MFA [46, 47].

## Conclusions

In this study, the design of the ^13^C-MFA experiment was optimized by computer simulations, which showed that 100% [2-^13^C]ethanol was the best carbon source for the precise estimation of metabolic fluxes through the TCA cycle and PP pathway. The ^13^C-MFA result revealed that the metabolic flux distribution was totally rewired in the ethanol culture from that of glucose. Estimates of the ATP balance from the ^13^C-MFA data suggested that larger amounts of excess ATP was produced from ethanol via increased oxidative phosphorylation. The finding was confirmed by the intracellular ATP level under ethanol-assimilating condition being similarly higher than glucose. These results suggest that the excess ATP regeneration via the activation of oxidative phosphorylation was a mechanism responsible for the SAM overproduction under the ethanol-assimilating conditions, which also provided general knowledge for the development of *S. cerevisiae* cell factories for the cytosolic acetyl-CoA-derived products.

## Additional file

**Additional file 1.** Additional tables and figures.
