# Supplementary material for: 13C-metabolic flux analysis of ethanol-assimilating Saccharomyces cerevisiae for S-adenosyl-l-methionine production
Source: Microb Cell Fact. 2018 May 31;17:82. doi: 10.1186/s12934-018-0935-6 (PMC5977476; doi:10.1186/s12934-018-0935-6)
Supplement: Supplementary file 1 — Additional file 1. Additional tables and figures. [file 12934_2018_935_MOESM1_ESM.docx]

**Table S1.** Metabolic network model for ^13^C-MFA.

| Flux_number | Reaction | Carbon atom transitions |
| --- | --- | --- |
| r1 | EtOH --> AcAl | AB --> AB |
| r2 | AcAl --> Ac | AB --> AB |
| r3 | Ac --> AcCOA_c | AB --> AB |
| r4 | AcCOA_c --> AcCOA_m | AB --> AB |
| r5 | Oxa_m + AcCOA_m --> IsoCit | ABCD + EF --> DCBFEA |
| r6 | IsoCit --> aKG + CO_2__in | ABCDEF --> ABCDE + F |
| r7 | IsoCit --> Suc + Glxy | ABCDEF --> EDCF + AB |
| r8 | Glxy + AcCOA_c --> Oxa_c | AB + CD --> ABDC |
| r9 | aKG --> Suc + CO_2__in | ABCDE --> BCDE + A |
| r10 | Suc --> Mal | ABCD --> ABCD |
| r11 | Mal --> Suc | ABCD --> ABCD |
| r12 | Mal --> Oxa_m | ABCD --> ABCD |
| r13 | Oxa_m --> Mal | ABCD --> ABCD |
| r14 | Oxa_m --> Oxa_c | ABCD --> ABCD |
| r15 | Oxa_c --> Oxa_m | ABCD --> ABCD |
| r16 | Oxa_c --> PEP + CO_2__in | ABCD --> ABC + D |
| r17 | PEP --> Pyr_c | ABC --> ABC |
| r18 | Pyr_c --> Pyr_m | ABC --> ABC |
| r19 | Pyr_c --> AcAl + CO_2__in | ABC --> BC + A |
| r20 | Pyr_m --> AcCOA_m + CO_2__in | ABC --> BC + A |
| r21 | Pyr_c + CO_2__in --> Oxa_c | ABC + D --> ABCD |
| r22 | Mal --> Pyr_m + CO_2__in | ABCD --> ABC + D |
| r23 | PGA --> PEP | ABC --> ABC |
| r24 | PEP --> PGA | ABC --> ABC |
| r25 | GAP --> PGA | ABC --> ABC |
| r26 | PGA --> GAP | ABC --> ABC |
| r27 | DHAP --> GAP | ABC --> ABC |
| r28 | GAP --> DHAP | ABC --> ABC |
| r29 | F6P --> DHAP + GAP | ABCDEF --> CBA + DEF |
| r30 | DHAP + GAP --> F6P | CBA + DEF --> ABCDEF |
| r31 | G6P --> F6P | ABCDEF --> ABCDEF |
| r32 | F6P --> G6P | ABCDEF --> ABCDEF |
| r33 | Glc --> G6P | ABCDEF --> ABCDEF |
| r34 | G6P --> 6PG | ABCDEF --> ABCDEF |
| r35 | 6PG --> Ru5P + CO_2__in | ABCDEF --> BCDEF + A |
| r36 | Ru5P --> R5P | ABCDE --> ABCDE |
| r37 | R5P --> Ru5P | ABCDE --> ABCDE |
| r38 | Ru5P --> Xu5P | ABCDE --> ABCDE |
| r39 | Xu5P --> Ru5P | ABCDE --> ABCDE |
| r40 | R5P + Xu5P --> S7P + GAP | ABCDE + FGHIJ --> FGABCDE + HIJ |
| r41 | GAP + S7P --> R5P + Xu5P | HIJ + FGABCDE --> FGHIJ + ABCDE |
| r42 | GAP + S7P --> F6P + E4P | ABC + DEFGHIJ --> DEFABC + GHIJ |
| r43 | E4P + F6P --> S7P + GAP | GHIJ + DEFABC --> DEFGHIJ + ABC |

**Table S1** Metabolic network model for ^13^C-MFA. (Continued)

| Flux_number | Reaction | Carbon atom transitions |
| --- | --- | --- |
| r44 | E4P + Xu5P --> F6P + GAP | ABCD + EFGHI --> EFABCD + GHI |
| r45 | GAP + F6P --> Xu5P + E4P | GHI + EFABCD --> EFGHI + ABCD |
| r46 | Oxa_c --> Thr | ABCD --> ABCD |
| r47 | PGA --> Ser | ABC --> ABC |
| r48 | Ser --> Gly + CO_2__in | ABC --> AB + C |
| r49 | Glxy --> Gly | AB --> AB |
| r50 | Pyr_c --> pre_Ala | ABC --> ABC |
| r51 | Pyr_m --> pre_Ala | ABC --> ABC |
| r52 | AcAl --> EtOH_ex |  |
| r53 | G6P --> [Biomass] |  |
| r54 | AcCOA_c --> [Biomass] |  |
| r55 | Gly --> [Biomass] |  |
| r56 | Ser --> [Biomass] |  |
| r57 | Oxa_c --> [Biomass] |  |
| r58 | R5P --> [Biomass] |  |
| r59 | E4P --> [Biomass] |  |
| r60 | GAP --> [Biomass] |  |
| r61 | PGA --> [Biomass] |  |
| r62 | PEP --> [Biomass] |  |
| r63 | pre_Ala --> [Biomass] |  |
| r64 | Pyr_m --> [Biomass] |  |
| r65 | AcCOA_m --> [Biomass] |  |
| r66 | aKG --> [Biomass] |  |
| r67 | Thr --> [Biomass] |  |
| r68 | Oxa_c --> [SAM] |  |
| r69 | Mal --> [Malate] |  |
| r70 | Ac --> [Acetate] |  |
| r71 | Pyr_c --> [Lactate] |  |
| r72 | Pyr_c --> [Pyrvate] |  |
| r73 | IsoCit --> [Citrate] |  |
| r74 | Suc --> [Succinate] |  |
| r75 | DHAP --> [Glycerol] |  |
| r76 | CO_2_ --> CO_2__in | A --> A |
| r77 | CO_2__in --> CO_2__ex | A --> A |
| r78 | IsoCit --> Oxa_m + AcCOA_m | DCBFEA --> ABCD + EF |

CO_2__ex, extracellular CO_2_; CO_2__in, intracellular CO_2_; CO_2_, carbon dioxide; pre_Ala, precursor of alanine.

**Table S2** Biosynthesis pathways towards the amino acids for ^13^C-MFA.

| Amino acids | Reactions | Carbon atom transitions |
| --- | --- | --- |
| Ala57 | pre_Ala --> Ala57 | ABC --> ABC |
| Ala85 | pre_Ala --> Ala85 | ABC --> BC |
| Asp57 | Oxa_c --> Asp57 | ABCD --> ABCD |
| Asp85 | Oxa_c --> Asp85 | ABCD --> BCD |
| Asp302 | Oxa_c --> Asp302 | ABCD --> AB |
| Glu57 | aKG --> Glu57 | ABCDE --> ABCDE |
| Glu85 | aKG --> Glu85 | ABCDE --> BCDE |
| Glu159 | aKG --> Glu159 | ABCDE --> BCDE |
| Gly57 | Gly --> Gly57 | AB --> AB |
| Gly85 | Gly --> Gly85 | AB --> B |
| Ile85 | Oxa_c + Pyr_m --> Ile85 | ABCD + EFG --> BFGCD |
| Ile159 | Oxa_c + Pyr_m --> Ile159 | ABCD + EFG --> BFGCD |
| Leu85 | Pyr_m + Pyr_m + AcCOA_m --> Leu85 | ABC + DEF + GH --> HBCEF |
| Leu159 | Pyr_m + Pyr_m + AcCOA_m --> Leu159 | ABC + DEF + GH --> HBCEF |
| Phe57 | E4P + PEP + PEP --> Phe57 | ABCD + EFG + HIJ --> EFGIJABCD |
| Pro85 | aKG --> Pro85 | ABCDE --> BCDE |
| Pro159 | aKG --> Pro159 | ABCDE --> BCDE |
| Thr57 | Thr --> Thr57 | ABCD --> ABCD |
| Val57 | Pyr_m + Pyr_m --> Val57 | ABC + DEF --> DEBFC |
| Val85 | Pyr_m + Pyr_m --> Val85 | ABC + DEF --> EBFC |
| Val159 | Pyr_m + Pyr_m --> Val159 | ABC + DEF --> EBFC |

Table S3—S6 show cell composition of *S. cerevisiae.*

**Table S3** Macromolecular composition [in %(w/w)] [1].

|  | Carbon source | |
| --- | --- | --- |
|  | Glucose | Ethanol |
| Component |  |  |
| Protein | 40.9 | 44.0 |
| Lipid | 6.5 | 10.2 |
| Carbohydrate | 41.5 | 35.1 |
| RNA + DNA | 7.2 | 6.9 |

Lipid fraction was assumed to consisit only Triacyl glycerol.

Carbohydrate fraction was assumed to be a polymer of glucose-6P.

**Table S4** Amino acid composition [in %(mol/mol)] [1].

| Ala | 9.8 | Lys | 6.6 |
| --- | --- | --- | --- |
| Arg | 3.9 | Met | 1.1 |
| Asp/Asn | 9.3 | Phe | 3.8 |
| Cys | 0.1 | Pro | 4.2 |
| Glu/Gln | 15.5 | Ser | 5.3 |
| Gly | 8.9 | Thr | 5.6 |
| His | 1.9 | Trp | 0.7 |
| Ile | 5.9 | Tyr | 2.0 |
| Leu | 8.0 | Val | 7.3 |

**Table S5** Fatty acid composition [in %(w/w)] [2].

| 10:0 | 1.1 | 16:1 | 16.6 |
| --- | --- | --- | --- |
| 12:0 | 4.8 | 18:0 | 6.1 |
| 14:0 | 8.8 | 18:1 | 25.7 |
| 16:0 | 26.8 | 18:2 | 10.1 |

**Table S6** RNA, DNA monomer composition [in %(mol/mol)] [2].

| RNA |  | DNA |  |
| --- | --- | --- | --- |
| AMP | 23.3 | dAMP | 29.8 |
| GMP | 23.3 | dGMP | 20.2 |
| CMP | 30.6 | dCMP | 20.2 |
| UMP | 22.8 | dTMP | 29.8 |

**Table S7** The demand of precursor metabolites (in μmol/g_CDW_).

|  | Carbon source | |
| --- | --- | --- |
|  | Glucose | Ethanol |
| Compound |  |  |
| Glucose 6-phosphate | 2306 | 1950 |
| Ribose 5-phosphate | 297 | 293 |
| Erythrose 4-phosphate | 206 | 221 |
| Glyceraldehyde 3-phosphate | 80 | 126 |
| Phosphoglycerate/Glyoxylate (for glycine) | 0 | 308 |
| Phosphoglycerate/Oxaloacetate (for glycine) | 288 | 0 |
| Phosphoglycerate (for others) | 277 | 286 |
| Phosphoenolpyruvate | 391 | 419 |
| Oxaloacetate (for threonine) | 180 | 193 |
| Oxaloacetate (for others) | 641 | 674 |
| Pyruvate (for alanine) | 316 | 339 |
| Pyruvate (mitochondrial, for others) | 867 | 1269 |
| Acetyl-CoA (cytosolic) | 2242 | 3433 |
| Acetyl-CoA (mitochondrial) | 259 | 278 |
| α-ketoglutarate | 975 | 1045 |

**Table S8** Flux for biomass and products syntheses (in mmol/g_CDW_/h).

|  | Carbon source | |
| --- | --- | --- |
|  | Glucose^a^ | Ethanol |
| Reaction |  |  |
| G6P --> [Biomass] | 0.14 | 0.12 |
| AcCOA_c --> [Biomass] | 0.14 | 0.21 |
| Gly --> [Biomass] | 0.023 | 0.024 |
| Ser --> [Biomass] | 0.010 | 0.011 |
| Oxa_c --> [Biomass] | 0.039 | 0.040 |
| R5P --> [Biomass] | 0.018 | 0.018 |
| E4P --> [Biomass] | 0.012 | 0.013 |
| GAP --> [Biomass] | 0.0048 | 0.0075 |
| PGA --> [Biomass] | 0.00027 | 0.00029 |
| PEP --> [Biomass] | 0.024 | 0.025 |
| pre_Ala --> [Biomass] | 0.019 | 0.020 |
| Pyr_m --> [Biomass] | 0.072 | 0.076 |
| AcCOA_m --> [Biomass] | 0.016 | 0.017 |
| aKG --> [Biomass] | 0.059 | 0.063 |
| Thr --> [Biomass] | 0.011 | 0.012 |
| Oxa_c --> [SAM] | 0.000089 | 0.00027 |
| AcAl --> [Ethanol] | 0 | - |
| Mal --> [Malate] | 0.0036 | 0.0051 |
| Ac --> [Acetate] | 0.0049 | 0.029 |
| Pyr_c --> [Lactate] | 0.0059 | 0.0020 |
| Pyr_c --> [Pyrvate] | 0 | 0.00027 |
| IsoCit --> [Citrate] | 0.0013 | 0.00051 |
| Suc --> [Succinate] | 0.0042 | 0.0043 |
| DHAP --> [Glycerol] | 0.00035 | 0.00044 |

The flux values of the precursor to biomass and products (SAM, ethanol, glycerol, lactate, acetate, citrate, malate, pyruvate, and succinate) were calculated based on the each requirement amount shown in Table S7 and Table 1 using following equation.

Flux for biomass synthesis$=\frac{W\times D}{1000}$

*W* represents the demand of precursor metabolites for biomass synthesis (μmol-metabolite/g_CDW_) described in the Table S7. *D* represents dilution rate (h^−1^).

Flux for product synthesis$=\frac{\rho}{1000}$

*ρ* represents specific production rate (μmol/g_CDW_/h) described in the Table 1.

^a^ Data from previous study [3].

**Table S9** 95% confidence intervals of flux estimated by the computer simulation of ^13^C-MFA using 10 mixtures of non-labeled, [1-^13^C], [2-^13^C], and [U-^13^C]ethanol as carbon source.

|  |  |  | Non-labeled:[1-^13^C]:[2-^13^C]:[U-^13^C]ethanol | | | | |
| --- | --- | --- | --- | --- | --- | --- | --- |
| Reaction |  | Flux value | [10:0:0:0] | [5:5:0:0] | [5:0:5:0] | [5:0:0:5] | [0:10:0:0] |
| **Glycolysis** | |  |  |  |  |  |  |
| PGI | G6P <--> F6P | -14.7 | -68.6 – -4.7 | -39.7 – -4.7 | -36.8 – -4.7 | -27.1 – -4.7 | -26.5 – -4.7 |
| PFK, FBA | F6P <--> DHAP + GAP | -8.6 | -26.6 – -5.3 | -17.0 – -5.3 | -16.0 – -5.3 | -12.8 – -5.3 | -12.6– -5.3 |
| TPI | DHAP <--> GAP | -8.7 | -26.6 – -5.3 | -17.0 – -5.3 | -16.0 – -5.3 | -12.8 – -5.3 | -12.6 – -5.3 |
| PGK | GAP <--> PGA | -14.9 | -32.8 – -11.5 | -23.2 – -11.5 | -22.2 – -11.5 | -19.0 – -11.5 | -18.8 – -11.5 |
| ENO | PGA <--> PEP | -15.6 | -33.8 – -12.0 | -24.0 – -12.0 | -22.7 – -12.0 | -20.0 – -12.1 | -19.3 – -12.0 |
| PYK | PEP --> Pyr_c | 62.9 | 0.1 – 500.0 | 18.3 – 186.2 | 5.2 – 500.0 | 39.5 – 68.2 | 25.1 – 108.7 |
| **TCA cycle** | |  |  |  |  |  |  |
| CIT | Oxa_m + AcCOA_m <--> IsoCit | 64.4 | 46.2 – 68.0 | 56.0 – 68.0 | 57.3 – 68.0 | 60.0 – 67.9 | 60.8 – 68.0 |
| IDH | IsoCit --> aKG + CO2 | 34.2 | 2.5 – 45.1 | 19.0 – 42.3 | 19.3 – 41.8 | 28.8 – 44.1 | 27.8 – 42.1 |
| KGD | aKG --> Suc + CO2 | 31.7 | 0.0 – 42.6 | 16.5 – 39.8 | 16.8 – 39.4 | 26.3 – 41.6 | 25.3 – 39.6 |
| SDH, FUM | Suc <--> Mal | 61.7 | 43.6 – 65.3 | 53.3– 65.3 | 54.6 – 65.3 | 57.3 – 65.2 | 58.1 –65.3 |
| MDH | Mal <--> Oxa_m | 56.5 | 18.7 – 65.1 | 47.8 – 65.1 | 46.2 – 65.1 | 52.5 – 62.6 | 51.2 – 65.1 |
| **Glyoxylate shunt** | |  |  |  |  |  |  |
| ICL | IsoCit --> Suc + Glxy | 30.2 | 22.9 – 65.5 | 25.6 – 38.3 | 26.0 – 40.1 | 23.5– 31.7 | 25.9 – 33.3 |
| MLS | AcCOA_c + Glxy --> Oxa_c | 29.6 | 21.9 – 64.5 | 24.7 – 37.6 | 25.1 – 39.1 | 22.8 – 31.3 | 24.9 – 32.4 |
| **PP pathway** | |  |  |  |  |  |  |
| ZWF | G6P --> 6PG | 10.0 | 0.0 – 63.9 | 0.0 – 35.0 | 0.0 – 32.1 | 0.0 – 22.4 | 0.0 – 21.8 |
| GND | 6PG --> Ru5P + CO2 | 10.0 | 0.0 – 63.9 | 0.0 – 35.0 | 0.0 – 32.1 | 0.0– 22.4 | 0.0 – 21.8 |
| RKI | Ru5P <--> R5P | 4.0 | 0.6 – 22.0 | 0.6 – 12.3 | 0.6 – 11.3 | 0.6 – 8.1 | 0.6 – 7.9 |
| RPE | Ru5P <--> Xu5P | 6.0 | -0.6 – 42.0 | -0.6 – 22.7 | -0.6 – 20.7 | -0.6 – 14.3 | -0.6 – 13.9 |
| TKL1 | R5P + Xu5P <--> S7P + GAP | 3.3 | -0.1 – 21.3 | -0.1 – 11.6 | -0.1 – 10.6 | -0.1 – 7.4 | -0.1 – 7.2 |
| TKL2 | E4P + Xu5P <--> F6P + GAP | 2.7 | -0.1 – 21.3 | -0.1 – 11.6 | -0.1 – 10.6 | -0.1 – 7.4 | -0.1 – 7.2 |
| TAL | GAP + S7P <--> F6P + E4P | 3.3 | -0.6 – 20.7 | -0.6 – 11.1 | -0.6 – 10.1 | -0.6 – 6.9 | -0.6 – 6.7 |
| **Anaplerotic pathway** | |  |  |  |  |  |  |
| PYC | Pyr_c + CO2 --> Oxa_c | 60.0 | 0.0 – 500.0 | 14.7 – 178.3 | 4.6 – 500.0 | 39.4 – 65.8 | 24.8 – 106.6 |
| MAE | Mal --> Pyr_m + CO2 | 5.0 | 0.0 – 46.5 | 0.0 – 7.5 | 0.0 – 9.6 | 1.3 – 7.0 | 0.0 – 7.4 |
| **Gluconeogenesis** | |  |  |  |  |  |  |
| PCK | Oxa_c --> PEP + CO2 | 79.6 | 13.1 – 500.0 | 34.6 – 199.2 | 20.6 – 500.0 | 52.8 – 88.3 | 42.3– 121.8 |
| **Pyruvate branchpoint** | |  |  |  |  |  |  |
| ADH | Ethanol --> AcAl | 100.0 |  |  |  |  |  |
| ALD | AcAl --> Ac | 102.0 | 100.0 – 142.6 | 100.0 – 106.0 | 100.0 – 110.8 | 100.0 – 105.0 | 100.0 – 105.6 |
| ACS | Ac --> AcCOA_c | 100.9 | 98.8 – 141.4 | 98.8 – 104.8 | 98.8 – 109.6 | 98.8 – 103.9 | 98.8 – 104.4 |
| PDC | Pyr_c --> AcAl + CO2 | 2.0 | 0.0 – 42.6 | 0.0 – 6.0 | 0.0 – 10.8 | 0.0 – 5.0 | 0.0 – 5.6 |
| PDA | Pyr_m --> AcCOA_m + CO2 | 2.0 | 0.0 – 42.6 | 0.0 – 3.9 | 0.0 – 5.9 | 0.0 – 3.0 | 0.0 – 3.6 |
| **Intracellular Transport** | |  |  |  |  |  |  |
| ACCOAt | AcCOA_c --> AcCOA_m | 63.1 | 26.1 – 68.7 | 54.1 – 68.7 | 53.5 – 68.7 | 59.7 – 68.5 | 58.6 – 68.7 |
| OXAt | Oxa_m <--> Oxa_c | -7.9 | -49.4 – -2.9 | -10.4 – -2.9 | -12.5 – -2.9 | -9.9 – -4.3 | -10.3– -2.9 |
| PYRt | Pyr_c --> Pyr_m | 0.8 | 0.0 – 46.5 | 0.0 – 11.4 | 0.0 – 9.1 | 0.0 – 3.6 | 0.0 – 7.2 |

**Table S9** 95% confidence intervals of flux estimated by the computer simulation of ^13^C-MFA using 10 mixtures of non-labeled, [1-^13^C], [2-^13^C], and [U-^13^C]ethanol as carbon source. (Continued)

|  |  |  | Non-labeled:[1-^13^C]:[2-^13^C]:[U-^13^C]ethanol | | | | |
| --- | --- | --- | --- | --- | --- | --- | --- |
| Reaction |  | Flux value | [10:0:0:0] | [5:5:0:0] | [5:0:5:0] | [5:0:0:5] | [0:10:0:0] |
| **Glycolysis** | |  |  |  |  |  |  |
| PGI | G6P <--> F6P | -14.7 | -68.6 – -4.7 | -39.7 – -4.7 | -36.8 – -4.7 | -27.1 – -4.7 | -26.5 – -4.7 |
| PFK  FBA | F6P <--> DHAP + GAP | -8.6 | -26.6 – -5.3 | -17.0 – -5.3 | -16.0 – -5.3 | -12.8 – -5.3 | -12.6 – -5.3 |
| TPI | DHAP <--> GAP | -8.7 | -26.6 – -5.3 | -17.0 – -5.3 | -16.0 – -5.3 | -12.8 – -5.3 | -12.6 – -5.3 |
| PGK | GAP <--> PGA | -14.9 | -32.8 – -11.5 | -23.2 – -11.5 | -22.2 – -11.5 | -19.0 – -11.5 | -18.8 – -11.5 |
| ENO | PGA <--> PEP | -15.6 | -33.8 – -12.0 | -24.0 – -12.0 | -22.7 – -12.0 | -20.0 – -12.1 | -19.3 – -12.0 |
| PYK | PEP --> Pyr_c | 62.9 | 0.1 – 500.0 | 18.3 – 186.2 | 5.2 – 500.0 | 39.5 – 68.2 | 25.1 – 108.7 |
| **TCA cycle** | |  |  |  |  |  |  |
| CIT | Oxa_m + AcCOA_m <--> IsoCit | 64.4 | 46.2 – 68.0 | 56.0 – 68.0 | 57.3 – 68.0 | 60.0 – 67.9 | 60.8 – 68.0 |
| IDH | IsoCit --> aKG + CO2 | 34.2 | 2.5 – 45.1 | 19.0 – 42.3 | 19.3 – 41.8 | 28.8 – 44.1 | 27.8 – 42.1 |
| KGD | aKG --> Suc + CO2 | 31.7 | 0.0 – 42.6 | 16.5 – 39.8 | 16.8 – 39.4 | 26.3 – 41.6 | 25.3 – 39.6 |
| SDH  FUM | Suc <--> Mal | 61.7 | 43.6 – 65.3 | 53.3 – 65.3 | 54.6 – 65.3 | 57.3 – 65.2 | 58.1 – 65.3 |
| MDH | Mal <--> Oxa_m | 56.5 | 18.7 – 65.1 | 47.8 – 65.1 | 46.2 – 65.1 | 52.5 – 62.6 | 51.2 – 65.1 |
| **Glyoxylate shunt** | |  |  |  |  |  |  |
| ICL | IsoCit --> Suc + Glxy | 30.2 | 22.9 – 65.5 | 25.6 – 38.3 | 26.0 – 40.1 | 23.5 – 31.7 | 25.9 – 33.3 |
| MLS | AcCOA_c + Glxy --> Oxa_c | 29.6 | 21.9 – 64.5 | 24.7 – 37.6 | 25.1 – 39.1 | 22.8 – 31.3 | 24.9 – 32.4 |
| **PP pathway** | |  |  |  |  |  |  |
| ZWF | G6P --> 6PG | 10.0 | 0.0 – 63.9 | 0.0 – 35.0 | 0.0 – 32.1 | 0.0 – 22.4 | 0.0 – 21.8 |
| GND | 6PG --> Ru5P + CO2 | 10.0 | 0.0 – 63.9 | 0.0 – 35.0 | 0.0 – 32.1 | 0.0 – 22.4 | 0.0 – 21.8 |
| RKI | Ru5P <--> R5P | 4.0 | 0.6 – 22.0 | 0.6 – 12.3 | 0.6 – 11.3 | 0.6 – 8.1 | 0.6 – 7.9 |
| RPE | Ru5P <--> Xu5P | 6.0 | -0.6 – 42.0 | -0.6 – 22.7 | -0.6 – 20.7 | -0.6 – 14.3 | -0.6 – 13.9 |
| TKL1 | R5P + Xu5P <--> S7P + GAP | 3.3 | -0.1 – 21.3 | -0.1 – 11.6 | -0.1 – 10.6 | -0.1 – 7.4 | -0.1 – 7.2 |
| TKL2 | E4P + Xu5P <--> F6P + GAP | 2.7 | -0.1 – 21.3 | -0.1 – 11.6 | -0.1 – 10.6 | -0.1 – 7.4 | -0.1 – 7.2 |
| TAL | GAP + S7P <--> F6P + E4P | 3.3 | -0.6 – 20.7 | -0.6 – 11.1 | -0.6 – 10.1 | -0.6 – 6.9 | -0.6 – 6.7 |
| **Anaplerotic pathway** | |  |  |  |  |  |  |
| PYC | Pyr_c + CO2 --> Oxa_c | 60.0 | 0.0 – 500.0 | 14.7 – 78.3 | 4.6 –500.0 | 39.4 – 65.8 | 24.8 – 106.6 |
| MAE | Mal --> Pyr_m + CO2 | 5.0 | 0.0 – 46.5 | 0.0 – 7.5 | 0.0 – 9.6 | 1.3 – 7.0 | 0.0 – 7.4 |
| **Gluconeogenesis** | |  |  |  |  |  |  |
| PCK | Oxa_c --> PEP + CO2 | 79.6 | 13.1 – 500.0 | 34.6 – 199.2 | 20.6 – 500.0 | 52.8 – 88.3 | 42.3 – 121.8 |
| **Pyruvate branchpoint** | |  |  |  |  |  |  |
| ADH | Ethanol --> AcAl | 100.0 |  |  |  |  |  |
| ALD | AcAl --> Ac | 102.0 | 100.0 – 142.6 | 100.0 – 106.0 | 100.0 – 110.8 | 100.0 – 105.0 | 100.0 – 105.6 |
| ACS | Ac --> AcCOA_c | 100.9 | 98.8 – 141.4 | 98.8 – 104.8 | 98.8 – 109.6 | 98.8 – 103.9 | 98.8 – 104.4 |
| PDC | Pyr_c --> AcAl + CO2 | 2.0 | 0.0 – 42.6 | 0.0 – 6.0 | 0.0 – 10.8 | 0.0 – 5.0 | 0.0 – 5.6 |
| PDA | Pyr_m --> AcCOA_m + CO2 | 2.0 | 0.0 – 42.6 | 0.0 – 3.9 | 0.0 – 5.9 | 0.0 – 3.0 | 0.0 – 3.6 |
| **Intracellular Transport** | |  |  |  |  |  |  |
| ACCOAt | AcCOA_c --> AcCOA_m | 63.1 | 26.1 – 68.7 | 54.1 – 68.7 | 53.5 – 68.7 | 59.7 – 68.5 | 58.6 – 68.7 |
| OXAt | Oxa_m <--> Oxa_c | -7.9 | -49.4 – -2.9 | -10.4 – -2.9 | -12.5 – -2.9 | -9.9 – -4.3 | -10.3 – -2.9 |
| PYRt | Pyr_c --> Pyr_m | 0.8 | 0.0 – 46.5 | 0.0 – 11.4 | 0.0 – 9.1 | 0.0 – 3.6 | 0.0 – 7.2 |

**Table S10** Experimentally measured and simulated ^13^C-enrichment of proteinogenic amino acids on ethanol (Dilution rate: 0.06 h^−1^).

| Amino acid | Fragment | Origin^a^ | ^13^C-enrichment | | | | | | | | | |
| --- | --- | --- | --- | --- | --- | --- | --- | --- | --- | --- | --- | --- |
|  |  |  | m_0_ | m_1_ | m_2_ | m_3_ | m_4_ | m_5_ | m_6_ | m_7_ | m_8_ | m_9_ |
| Ala | M-57 | Exp | 0.012 | 0.024 | 0.687 | 0.276 |  |  |  |  |  |  |
|  |  | Sim | 0.002 | 0.024 | 0.681 | 0.284 |  |  |  |  |  |  |
| Ala | M-85 | Exp | 0.010 | 0.032 | 0.958 |  |  |  |  |  |  |  |
|  |  | Sim | 0.005 | 0.032 | 0.954 |  |  |  |  |  |  |  |
| Asx^b^ | M-57 | Exp | 0.010 | 0.017 | 0.452 | 0.503 | 0.018 |  |  |  |  |  |
|  |  | Sim | 0.001 | 0.014 | 0.470 | 0.488 | 0.017 |  |  |  |  |  |
| Asx | M-85 | Exp | 0.013 | 0.021 | 0.710 | 0.256 |  |  |  |  |  |  |
|  |  | Sim | 0.001 | 0.022 | 0.726 | 0.242 |  |  |  |  |  |  |
| Asx | M302 | Exp | 0.021 | 0.687 | 0.292 |  |  |  |  |  |  |  |
|  |  | Sim | 0.010 | 0.699 | 0.285 |  |  |  |  |  |  |  |
| Glx^c^ | M-57 | Exp | 0.000 | 0.002 | 0.027 | 0.696 | 0.263 | 0.012 |  |  |  |  |
|  |  | Sim | 0.000 | 0.002 | 0.028 | 0.693 | 0.258 | 0.008 |  |  |  |  |
| Glx | M-85 | Exp | 0.001 | 0.004 | 0.036 | 0.926 | 0.033 |  |  |  |  |  |
|  |  | Sim | 0.000 | 0.002 | 0.036 | 0.920 | 0.031 |  |  |  |  |  |
| Glx | M-159 | Exp | 0.000 | 0.006 | 0.036 | 0.918 | 0.039 |  |  |  |  |  |
|  |  | Sim | 0.000 | 0.002 | 0.036 | 0.920 | 0.031 |  |  |  |  |  |
| Gly | M-57 | Exp | 0.023 | 0.713 | 0.265 |  |  |  |  |  |  |  |
|  |  | Sim | 0.011 | 0.714 | 0.268 |  |  |  |  |  |  |  |
| Gly | M-85 | Exp | 0.028 | 0.972 |  |  |  |  |  |  |  |  |
|  |  | Sim | 0.019 | 0.976 |  |  |  |  |  |  |  |  |
| Ile | M-85 | Exp | 0.017 | 0.004 | 0.011 | 0.040 | 0.699 | 0.229 |  |  |  |  |
|  |  | Sim | 0.000 | 0.000 | 0.006 | 0.045 | 0.701 | 0.231 |  |  |  |  |
| Ile | M-159 | Exp | 0.016 | 0.002 | 0.013 | 0.043 | 0.697 | 0.229 |  |  |  |  |
|  |  | Sim | 0.000 | 0.000 | 0.006 | 0.045 | 0.701 | 0.231 |  |  |  |  |
| Leu | M-85 | Exp | 0.020 | 0.007 | 0.001 | 0.011 | 0.062 | 0.899 |  |  |  |  |
|  |  | Sim | 0.000 | 0.000 | 0.000 | 0.012 | 0.070 | 0.899 |  |  |  |  |
| Leu | M-159 | Exp | 0.018 | 0.006 | 0.002 | 0.014 | 0.073 | 0.887 |  |  |  |  |
|  |  | Sim | 0.000 | 0.000 | 0.000 | 0.012 | 0.070 | 0.899 |  |  |  |  |
| Phe | M-57 | Exp | 0.016 | 0.001 | 0.000 | 0.001 | 0.005 | 0.027 | 0.283 | 0.425 | 0.209 | 0.032 |
|  |  | Sim | 0.000 | 0.000 | 0.000 | 0.000 | 0.004 | 0.032 | 0.278 | 0.411 | 0.208 | 0.035 |
| Pro | M-85 | Exp | 0.014 | 0.012 | 0.036 | 0.916 | 0.022 |  |  |  |  |  |
|  |  | Sim | 0.000 | 0.002 | 0.036 | 0.920 | 0.031 |  |  |  |  |  |
| Pro | M-159 | Exp | 0.015 | 0.010 | 0.036 | 0.914 | 0.026 |  |  |  |  |  |
|  |  | Sim | 0.000 | 0.002 | 0.036 | 0.920 | 0.031 |  |  |  |  |  |
| Thr | M-57 | Exp | 0.015 | 0.014 | 0.485 | 0.474 | 0.012 |  |  |  |  |  |
|  |  | Sim | 0.001 | 0.014 | 0.470 | 0.488 | 0.017 |  |  |  |  |  |
| Thr | M-85 | Exp | 0.014 | 0.019 | 0.742 | 0.225 |  |  |  |  |  |  |
|  |  | Sim | 0.001 | 0.022 | 0.726 | 0.242 |  |  |  |  |  |  |
| Val | M-85 | Exp | 0.012 | 0.003 | 0.009 | 0.055 | 0.921 |  |  |  |  |  |
|  |  | Sim | 0.000 | 0.000 | 0.011 | 0.061 | 0.910 |  |  |  |  |  |
| Val | M-159 | Exp | 0.012 | 0.003 | 0.014 | 0.066 | 0.906 |  |  |  |  |  |
|  |  | Sim | 0.000 | 0.000 | 0.011 | 0.061 | 0.910 |  |  |  |  |  |

^a^ Exp, experimentally measured by GC-MS analysis; Sim, computationally simulated.

^b^ ASX, Asp and Asn.

^c^ Glx, Glu and Gln.

**Table S11** Esimated best-fit values of metabolic fluxes from ^13^C-MFA (in mmol/g_CDW_/h).

|  | Carbon source | |
| --- | --- | --- |
|  | Glucose^a^ | Ethanol |
| Reaction |  |  |
| Glc --> G6P | 0.65 | - |
| EtOH --> AcAl | - | 2.5 |
| AcAl --> Ac | 0.21 | 2.5 |
| Ac --> AcCOA_c | 0.21 | 2.5 |
| AcCOA_c --> AcCOA_m | 0.060 | 1.7 |
| Oxa_m + AcCOA_m --> IsoCit | 0.40 | 1.7 |
| IsoCit --> aKG + CO2_in | 0.39 | 1.1 |
| IsoCit --> Suc + Glxy | 0.013 | 0.62 |
| AcCOA_c + Glxy --> Oxa_c | 0.013 | 0.61 |
| aKG --> Suc + CO2_in | 0.33 | 1.0 |
| Suc <--> Mal | 0.34 | 1.6 |
| Mal <--> Oxa_m | 0.27 | 1.6 |
| Oxa_m <--> Oxa_c | −0.13 | −0.10 |
| Oxa_c --> PEP + CO2_in | - | 1.4 |
| PEP --> Pyr_c | 0.77 | 1.0 |
| Pyr_c --> Pyr_m | 0.39 | 0.104 |
| Pyr_c --> AcAl + CO2_in | 0.21 | 0.0 |
| Pyr_m --> AcCOA_m + CO2_in | 0.36 | 0.038 |
| Pyr_c + CO2_in --> Oxa_c | 0.17 | 0.91 |
| Mal --> Pyr_m + CO2_in | 0.061 | 0.031 |
| PGA <--> PEP | 0.80 | −0.32 |
| GAP <--> PGA | 0.83 | −0.30 |
| DHAP <--> GAP | 0.35 | −0.14 |
| F6P <--> DHAP + GAP | 0.35 | −0.14 |
| G6P <--> F6P | 0.077 | −0.14 |
| G6P --> 6PG | 0.44 | 0.024 |
| 6PG --> Ru5P + CO2_in | 0.44 | 0.024 |
| Ru5P <--> R5P | 0.16 | 0.024 |
| Ru5P <--> Xu5P | 0.28 | 0.0 |
| R5P + Xu5P <--> S7P + GAP | 0.14 | 0.0066 |
| GAP + S7P <--> F6P + E4P | 0.14 | 0.0066 |
| E4P + Xu5P <--> F6P + GAP | 0.13 | −0.0066 |
| Oxacyt --> preThr | 0.012 | 0.012 |
| preThr --> preGly + AcAl | 0.0011 | - |
| PGA --> preSer | 0.033 | 0.022 |
| preSer --> preGly + CO2_in | 0.022 | 0.011 |
| Glxy --> preGly | - | 0.014 |
| Pyrcyt --> preAla | 0.0030 | 0.0 |
| Pyrmit --> preAla | 0.016 | 0.020 |

^a^ Data from previous study [3].


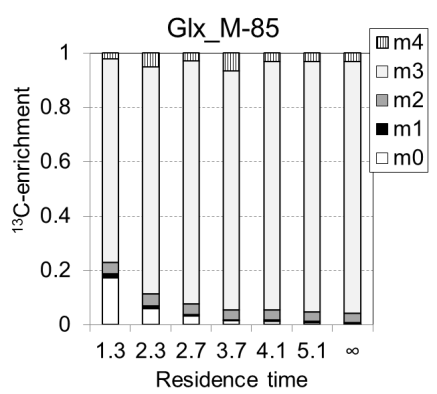

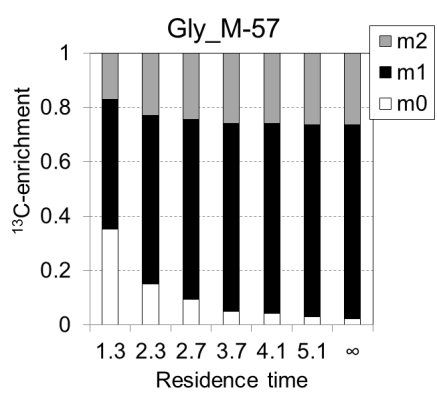

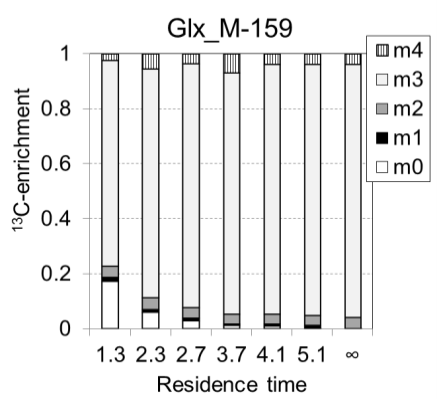

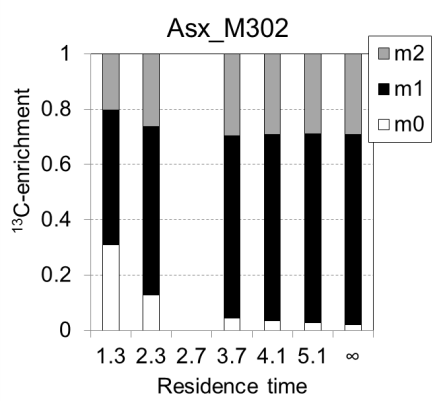
**
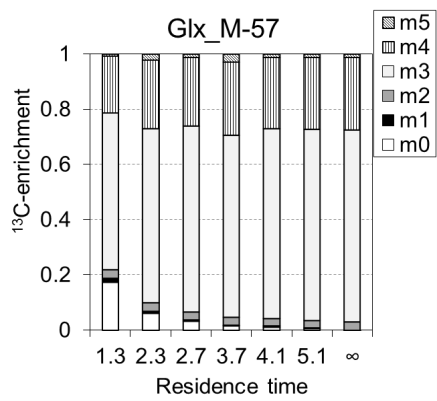
**
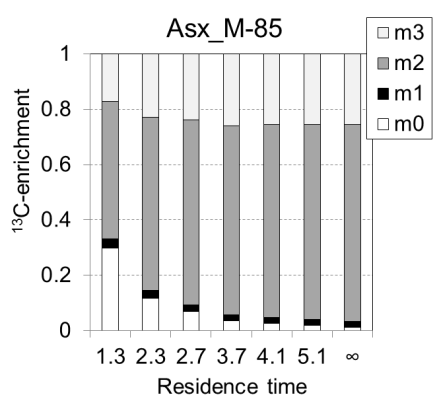
**
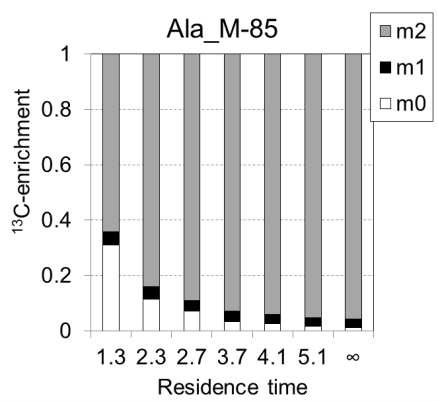
**
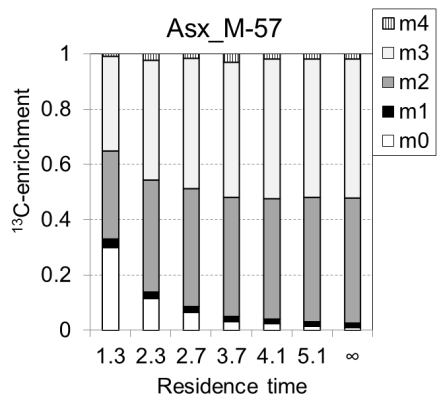
**
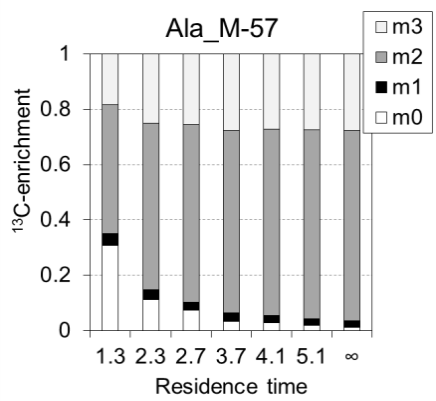
Fig. S1.** Time courses of ^13^C-enrichment of proteinogenic amino acids after feeding ^13^C-labeled ethanol in dilution rate 0.06 h^−1^ condition.

Residence time represents the moment which have started feeding ^13^C-labeled ethanol as zero. M−57, M−85, M−159 and M302 of each amino acid indicate fragment ion. m*_n_* represents the mass isotopomer containing *n* ^13^C atoms.


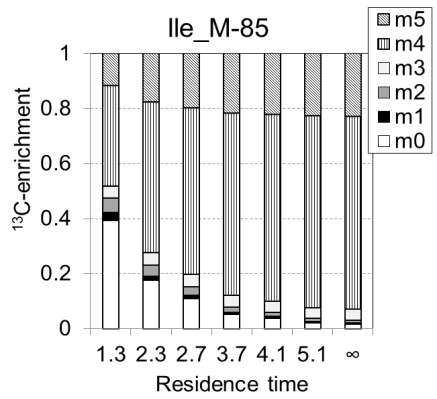

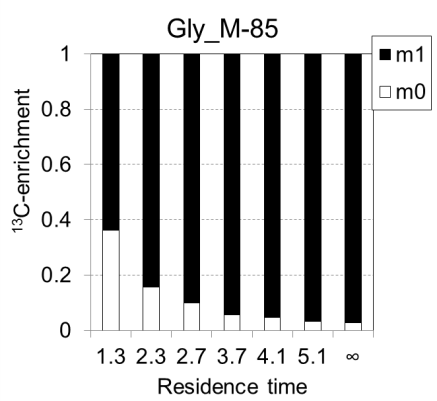

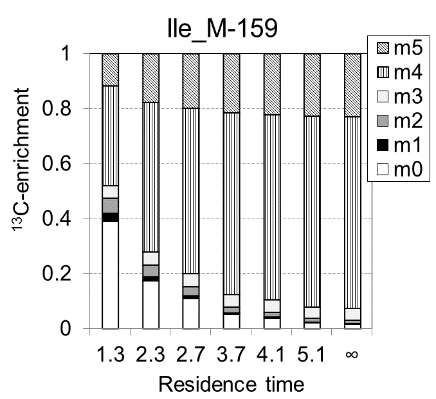

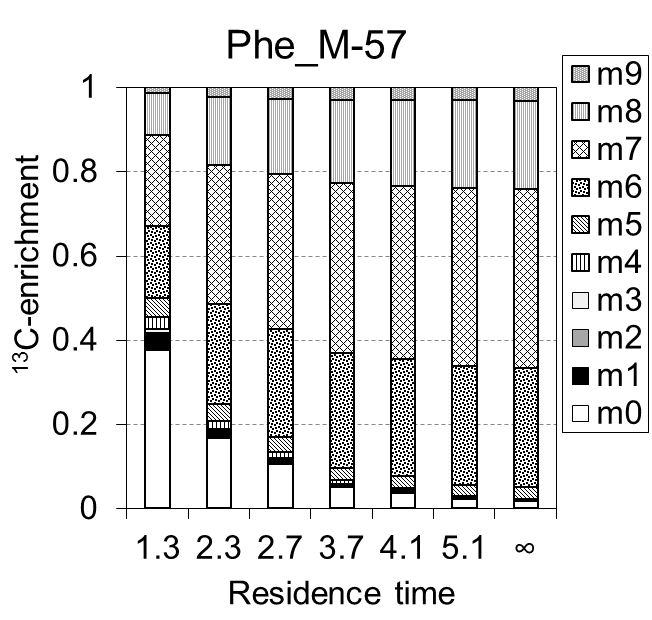

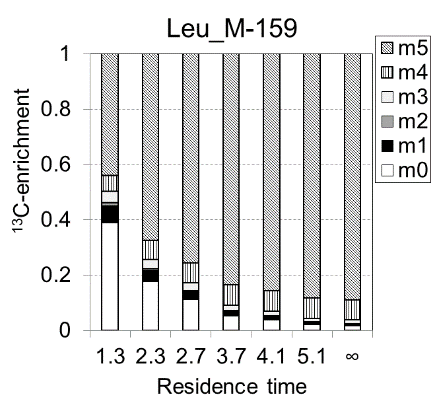

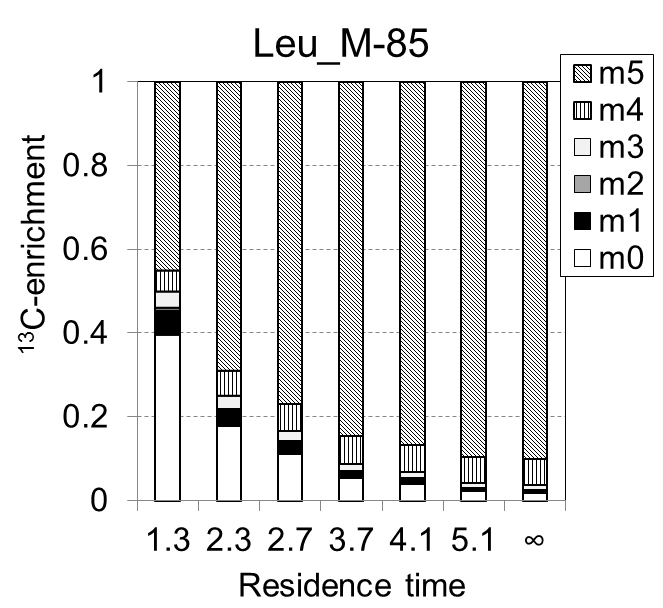

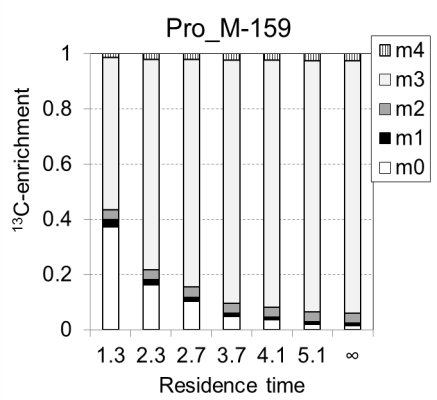

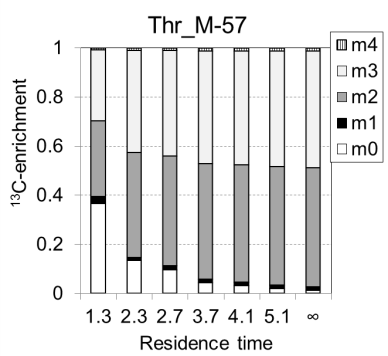
**
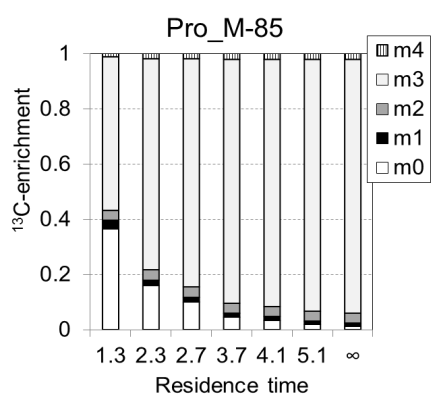
**

**Fig. S1.** Time courses of ^13^C-enrichment of proteinogenic amino acids after feeding ^13^C-labeled ethanol in dilution rate 0.06 h^−1^ condition.

Residence time represents the moment which have started feeding ^13^C-labeled ethanol as zero. M−57, M−85, M−159 and M302 of each amino acid indicate fragment ion. m*_n_* represents the mass isotopomer containing *n* ^13^C atoms. (Continued)


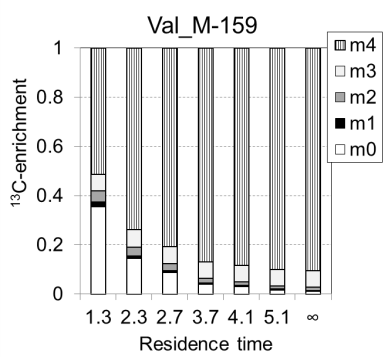

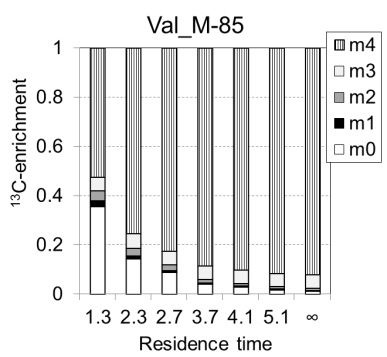

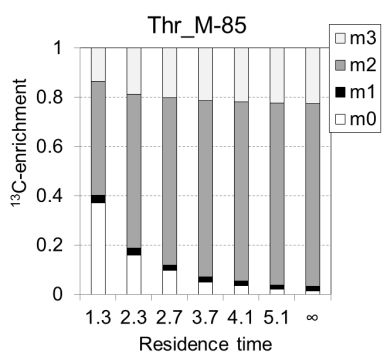


**Fig. S1.** Time courses of ^13^C-enrichment of proteinogenic amino acids after feeding ^13^C-labeled ethanol in dilution rate 0.06 h^−1^ condition.

Residence time represents the moment which have started feeding ^13^C-labeled ethanol as zero. M−57, M−85, M−159 and M302 of each amino acid indicate fragment ion. m*_n_* represents the mass isotopomer containing *n* ^13^C atoms. (Continued)

**Reference**

1. Stückrath I, Lange HC, Kötter P, van Gulik WM, Entian KD, Heijnen JJ. Characterization of null mutants of the glyoxylate cycle and gluconeogenic enzymes in *S. cerevisiae* through metabolic network modeling verified by chemostat cultivation. Biotechnol Bioeng. 2002;77(1):61−72.

2. Förster J, Famili I, Fu P, Palsson BØ, Nielsen J. Genome-scale reconstruction of the *Saccharomyces cerevisiae* metabolic network. Genome Res. 2003;13(2):244−53.

3. Hayakawa K, Kajihata S, Matsuda F, Shimizu H. ^13^C-metabolic flux analysis in *S*-adenosyl-l-methionine production by *Saccharomyces cerevisiae*. J Biosci Bioeng. 2015;120:532–8.
